# Quantifying human gray matter microstructure using neurite exchange imaging (NEXI) and 300 mT/m gradients

**DOI:** 10.1162/imag_a_00104

**Published:** 2024-03-06

**Authors:** Quentin Uhl, Tommaso Pavan, Malwina Molendowska, Derek K. Jones, Marco Palombo, Ileana Ozana Jelescu

**Affiliations:** Department of Radiology, Lausanne University Hospital (CHUV), Lausanne, Switzerland; School of Biology and Medicine, University of Lausanne, Lausanne, Switzerland; Cardiff University Brain Research Imaging Centre (CUBRIC), Cardiff University, Cardiff, United Kingdom; School of Computer Science and Informatics, Cardiff University, Cardiff, United Kingdom

**Keywords:** diffusion MRI, biophysical models, cortex, human brain, exchange, in vivo

## Abstract

Biophysical models of diffusion tailored to quantify gray matter microstructure are gathering increasing interest. The two-compartment Neurite EXchange Imaging (NEXI) model has been proposed recently to account for neurites, extra-cellular space, and exchange across the cell membrane. NEXI parameter estimation requires multi-shell multi-diffusion time data and has so far only been implemented experimentally on animal data collected on a preclinical magnetic resonance imaging (MRI) set-up. In this work, the translation of NEXI to the human cortex in vivo was achieved using a 3 T Connectom MRI system with 300 mT/m gradients, that enables the acquisition of a broad range of b-values (0 – 7.5 ms/µm²) with a window covering short to intermediate diffusion times (20 – 49 ms) suitable for the characteristic exchange times (10 – 50 ms). Microstructure estimates of four model variants: NEXI, NEXI_dot_(its extension with the addition of a dot compartment), and their respective versions that correct for the Rician noise floor (NEXI_RM_and NEXI_dot,RM_) that particularly impacts high b-value signal, were compared. The reliability of estimates in each model variant was evaluated in synthetic and human in vivo data. In the latter, the intra-subject (scan-rescan) versus between-subjects variability of microstructure estimates was compared in the cortex. The better performance of NEXI_RM_highlights the importance of correcting for Rician bias in the NEXI model to obtain accurate estimates of microstructure parameters in the human cortex, and the sensitivity of the NEXI framework to individual differences in cortical microstructure. This application of NEXI in humans represents a significant step, unlocking new avenues for studying neurodevelopment, aging, and various neurodegenerative disorders.

## 1 Introduction

Quantifying microstructure features of the human cortex in vivo has the potential to significantly improve our understanding and management of neurological and psychiatric diseases, which are associated with cognitive, motor, and behavioral deficits (Illán-Gala et al., 2022;Nürnberger et al., 2017;Spotorno et al., 2022;Voldsbekk et al., 2022). Early diagnosis and effective treatment of these diseases remain a challenge, as their pathophysiology is not fully understood. Identifying the associated changes in the cortex microstructure could lead to a better understanding of the disease progression, earlier diagnoses and access to treatment, and help develop targeted therapies.

Diffusion-weighted magnetic resonance imaging (dMRI) can provide such an insight into the microstructure of the brain, by exploiting the sensitivity of the signal to the motion of water molecules within tissue. In particular, biophysical modeling of the dMRI signal aims to characterize the tissue microstructure by fitting an analytical model of the tissue described by its most relevant geometric and diffusion features (Alexander et al., 2019;Jelescu et al., 2020;Novikov et al., 2019,^2018^;Stanisz et al., 1997) to the measured signals.

There is already a wide variety of biophysical models of white matter, based on what is now commonly referred to as the “Standard Model” (Novikov et al., 2019) of non-exchanging compartments within which the diffusion displacement profile is Gaussian. However, recent studies indicate that the Standard Model does not hold in gray matter. At high b-values, the deviation of the directionally averaged signal in gray matter from the impermeable stick power-law$\bar{S}\propto b^{-1/2}$(McKinnon et al., 2017;Veraart, Fieremans, et al., 2016) prompted the hypotheses that other features such as the cell body or “soma” (Palombo et al., 2020,^2018^), inter-compartment exchange (Jelescu et al., 2022;Olesen et al., 2022;Veraart et al., 2018), and non-Gaussian diffusion within a compartment resulting from structural disorder (Henriques et al., 2019;Lee et al., 2020) should be accounted for. Indeed, in the cortex, most neurites are unmyelinated, so that the exchange of water between the intracellular and extracellular compartments may be significant for diffusion times that are longer than 20 ms (typical of the minimal diffusion time achievable on human MRI scanners). Additionally, the assumption of Gaussian diffusion within a given compartment may not hold in the presence of irregularities on length scales that are similar to the diffusion length, such as dendritic spines and neurite beading. Furthermore, the volume occupied by soma, in the gray matter, is approximately 10-20%, but negligible in white matter and therefore not currently included in white matter models.

As an extension of the Standard Model, the Soma And Neurite Density Imaging (SANDI) model (Palombo et al., 2020) incorporated the soma size and signal fraction in addition to neurite signal fraction, thereby enabling their joint estimation. However, as it does not account for inter-compartment exchange, the SANDI model is currently only applicable to data acquired within diffusion times shorter than 20 ms, for which the assumption of impermeable compartments is valid (Jelescu et al., 2020). As noted above, such diffusion times can only be achieved for very high b-values (up to 10 ms/μm²), on systems with ultra-strong gradients, such as preclinical scanners or human scanners with dedicated gradient sets (such as the Connectom scanner, 300 mT/m gradient amplitude) (Huang et al., 2021;Jones et al., 2018;Setsompop et al., 2013).

The Neurite Exchange Imaging (NEXI) model (Jelescu et al., 2022)—proposed in parallel byOlesen et al. (2022)as SMEX (Standard Model with EXchange)—was introduced recently to recognize and quantify water exchange across the neurite membrane. As such, NEXI is applicable on clinical-grade scanners because it does not necessarily require short diffusion times. NEXI models the neurites as a collection of randomly-oriented sticks—occupying a relative signal fraction*f*—where the intra-neurite diffusion is uniaxial with diffusivity$D_{i,∥}$. Moreover, given the quasi-uniform orientation-distribution of neurites in gray matter, the extra-neurite compartment is considered to be Gaussian isotropic with characteristic diffusivity$D_{e}$. The two compartments exchange with a characteristic time$t_{ex}$. NEXI models the total orientation-averaged signal as the sum of these two exchanging compartments. They are assumed to have the same transverse relaxation time, or*T*_2_. The soma are not explicitly modeled and the signal contribution arising from this compartment is most likely pooled with the signal contribution from the extra-cellular space in NEXI (Jelescu et al., 2022). Importantly, the experimental observation of decreasing signal with increasing diffusion times supports exchange as a dominant contributor to signal features over a soma compartment with restricted diffusion (Jelescu et al., 2022;Olesen et al., 2022), although accounting for soma improves the fit of the signal tail (highest b-values). Thus, if the available diffusion MRI data do not allow fitting a model with enough parameters to account for both exchange and soma, modeling exchange while neglecting soma can be justified for diffusion times*t*_d_longer than 20 ms. On the other hand, an extension of SMEX which also models the soma as a separate compartment (SANDIX—SANDI with eXchange) has been proposed and applied to ex-vivo preclinical data (Olesen et al., 2022). The stability of fitting such a large number of model parameters on human in-vivo data remains to be established.

The NEXI signal equation is a spherical mean of the kernel 𝒦, the anisotropic extension of the Kärger model of two well-mixed exchanging compartments in a barrier-limited regime (Fieremans et al., 2010;Jelescu et al., 2022;Kärger, 1985):

$${\bar{S}}_{NEXI}\left(p;q,t_{d}\right)=\int_{0}^{1}K\left(q,g,t_{d};p,n\right)d{\left(g.n\right)}^{2}$$ (1)

where$p=[t_{ex},D_{i,∥},D_{e},f]$are the microstructure parameters to fit,**n**are the neurite orientations, and*q*is the wave vector along direction**g**.

The assumption of the barrier-limited regime is supported if the characteristic time*t*_c_to reach the long-time diffusion limit in each compartment is shorter than the characteristic exchange time between compartments. In the case of infinitely long cylinders modeling the neurites, the radial plane is relevant for exchange across the membrane. In the case of neurites with a diameter d ~ 1 µm, the characteristic time in the intra-neurite space$t_{c,i}=\frac{d^{2}}{2D_{i}}\approx 0.25ms$and extra-neurite space$t_{c,e}=\frac{d^{2}}{2D_{e}}\frac{\pi }{f}\approx 7.5ms$at most—assuming the lower bound of*f*~0.3 (Fieremans et al., 2010). Both timescales are shorter than the exchange time reported in previous studies$t_{ex}>5\text{ ms}$. We note that the Kärger model assumption implies diffusion should be time-independent, while some time-dependence has been reported in a previous in vivo study of the human cortex (Lee et al., 2020), D(t) was weak and the long-time limit was reached for*t*_d_> 20 ms, which agrees with the experimental setting in the present study.

The aim of this study was to evaluate the feasibility and value of using the NEXI model and some of its variants for quantifying microstructural parameters in the human cortex in vivo.

To achieve this, we acquired multi-shell multi-diffusion time dMRI data in healthy human volunteers on a Connectom MRI system equipped with very strong (300 mT/m) gradients. The Connectom scanners are an important steppingstone in terms of hardware capabilities between preclinical MRI systems (with gradients >600 mT/m), and clinical MRI systems (with gradients ≤ 80 mT/m). They provide the opportunity for an initial translation of NEXI in human subjects by enabling the acquisition of the necessary broad range of b-values (0 – 7.5 ms/µm²) at diffusion times 20 – 49 ms, that are short enough to capture exchange processes with*t_ex_*= 10 - 50 ms, as previously reported for the brain cortex*in vivo*(Jelescu et al., 2022;Lee et al., 2020).

**Fig. 1.**
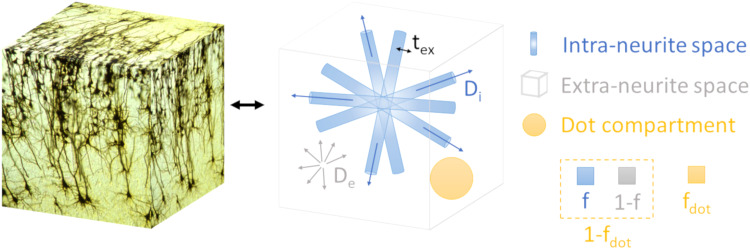
Sketch of relevant features and parameters in the NEXI_dot_model, the three-compartment variant of NEXI. The latter can be obtained by removing the dot compartment (in yellow) from the sketch.

Here, we compared NEXI-derived estimates in the human cortex to those obtained from its three-compartment variant, allowing for an extra “dot” compartment, filled with stationary water. This NEXI extension, referred to here as NEXI_dot_, has been proposed previously (Olesen et al., 2022) to explain the non-zero signal asymptote at high b-value ex vivo (Fig. 1). In the cerebellum, the presence of such a compartment has been shown in vivo (Tax et al., 2020), but its existence in the cortex remains unclear. This compartment’s stationary water signal does not decay with diffusion-weighting, thus yielding the NEXI_dot_signal attenuation equation:

$${\bar{S}}_{NEXI_{dot}}\left(p;q,t_{d}\right)=\left(1-f_{dot}\right).{\bar{S}}_{NEXI}\left(p;q,t_{d}\right)+f_{dot}$$ (2)

where$f_{dot}$is the stationary water fraction.

At high b-values, high spatial resolution, and moderate field strength, the diffusion-weighted signal magnitude is heavily affected by the Rician noise floor. The effect of this noise floor can be accounted for by considering the expectation value of the signal${\bar{S}}_{NEXI}\left(p;q,t_{d}\right)$given the normalized Rician noise level$\sigma =\frac{\sigma_{Signal}}{S_{b=0}}$. The NEXI signal equation corrected for the Rician Mean (RM) is:

$${\bar{S}}_{NEXI_{RM}}\left(p;q,t_{d},\sigma \right)=\sqrt{\frac{\pi }{2}}.\sigma .L_{1/2}\left(-\frac{1}{2}{\left(\frac{{\bar{S}}_{NEXI}\left(p;q,t_{d}\right)}{\sigma }\right)}^{2}\right)$$ (3)

where$L_{1/2}\left(x\right){=}_{1}F_{1}\left(-\frac{1}{2},1,x\right)$is the generalized Laguerre polynomial, expressed in terms of the confluent hypergeometric function of the first kind. This correction behaves like the identity function in cases where${\bar{S}}_{NEXI}\gg \sigma$and converges to$\sqrt{\frac{\pi }{2}}\sigma$, the Rician noise floor, for cases where${\bar{S}}_{NEXI}\leq \sigma$.

Similarly, the signal equation for the NEXI_dot,RM_model is:

$${\bar{S}}_{NEXI_{dot,RM}}\left(p;q,t_{d},\sigma \right)=\sqrt{\frac{\pi }{2}}.\sigma .L_{1/2}\left(-\frac{1}{2}{\left(\frac{{\bar{S}}_{NEXI_{dot}}\left(p;q,t_{d}\right)}{\sigma }\right)}^{2}\right)$$ (4)

We therefore compared NEXI and NEXI_dot_estimates to their respective RM-corrected counterparts.

Furthermore, we compared the estimates of*t_ex_*from the different model variants with the one from the Kärger model time-dependent kurtosis (Fieremans et al., 2010;Jelescu et al., 2022;Jensen & Helpern, 2010):

$$K_{KM}\left(t_{d}\right)=2\frac{t_{ex}}{t_{d}}\left[1-\frac{t_{ex}}{t_{d}}\left(1-e^{\frac{t_{d}}{t_{ex}}}\right)\right]$$ (5)

Finally, we estimated the repeatability and sensitivity of NEXI cortex microstructure estimates by comparing their intra-subject (scan-rescan) to inter-subject variability. Parameter spatial distribution across different brain regions was also evaluated in comparison with known distribution maps from postmortem histological staining.

## 2 Methods

### 2.1 Experimental

#### 2.1.1 Participants

The study was approved by the School of Psychology Ethics Committee at Cardiff University. Written informed consent was obtained from all participants. Data were acquired in four healthy adults (Age: 30.5 +/- 3.8 years; 2 M / 2F). Three participants were rescanned 2 days after the first scan.

#### 2.1.2 Data acquisition

All data were acquired on a Connectom MRI scanner, a modified 3 T MAGNETOM Skyra system fitted with a gradient coil capable of 300 mT/m (Siemens Healthcare, Erlangen, Germany). An anatomical reference was acquired using an MP-RAGE sequence (1-mm isotropic resolution, FOV = 256 x 256 mm^2^, 192 slices, TI/TR = 857/2300 ms). Diffusion-weighted images were acquired using a Pulsed Gradient Spin Echo Echo-Planar Imaging (PGSE EPI) sequence with b-values of 1 (13 directions), 2.5 (25 dir.), 4 (25 dir.), 6 (32 dir.), and 7.5 ms/µm² (65 dir.), at each of four diffusion times Δ = 20, 29, 39, and 49 ms, in addition to 15 b = 0 ms/µm² images per Δ. Other parameters were fixed: δ = 9 ms, TE/TR = 76 ms/3.7 s, FOV= 216 x 216 mm^2^, matrix: 120 x 120, 66 slices, 1.8-mm isotropic resolution, partial Fourier = 0.75, GRAPPA = 2, multiband = 2. The total dMRI scan time was 45 min.

#### 2.1.3 Data preprocessing

While each diffusion time was acquired in a separate scan, all multi-shell multi-diffusion time data (N = 700 volumes) were pooled together for pre-processing. Pre-processing included Marchenko-Pastur principal component analysis (MP-PCA) magnitude denoising (Veraart, Novikov, et al., 2016), Gibbs ringing correction (Kellner et al., 2016), distortion, and eddy current correction (Andersson & Sotiropoulos, 2016). A separate MP-PCA denoising of b = 0 and b = 1 ms/µm² images (N = 112 volumes) was used to extract an unbiased noisemap, σ, from high SNR data, to be used in the Rician mean correction (Eq. 3-4). For NEXI, data were averaged over directions (powder-average, using the arithmetic mean) and normalized by the mean value of the b = 0 ms/µm² volumes.

#### 2.1.4 Time-dependent kurtosis

DKI fitting (Jensen et al., 2005) was performed using a weighted linear least-squares algorithm implemented in Matlab (Veraart et al., 2013) to extract Mean Diffusivity (MD) and Mean Kurtosis (MK) for each diffusion time using b-values up to 2.5 ms/µm². K_KM_(t) (Eq. 5) was then fit to MK to yield an alternative estimation of*t_ex_*.

#### 2.1.5 ROI parcellation

Grey matter region of interests (ROIs) from the Desikan-Killiany-Tourville (DKT) atlas (Klein & Tourville, 2012) were segmented on the anatomical MPRAGE image using FastSurfer (Henschel et al., 2020) and transformed into diffusion native space using linear registration of distortion-corrected b = 0 ms/µm² images to MPRAGE images. The cortical ribbon was segmented by merging the gray matter ROIs obtained with the DKT atlas.

### 2.2 Simulations

Three separate datasets were generated.**Dataset 1**: Synthetic NEXI signals were generated usingEq. 1and the same diffusion times and b-values as the experimental acquisition. The ground truth parameters of each signal were randomly chosen within the following bounds with uniform probability distribution: [1 - 150] ms for*t*_ex_, [0.1 - 3.5] µm²/ms for the two diffusivities and [0.1 - 0.9] the fraction*f*, with the constraint that*D*_i_ >*D*_e_(Dhital et al., 2019;Howard et al., 2022;Kunz et al., 2018). Twenty Rician noise realizations were generated for each ground truth, assuming SNR = 34 at b = 0 ms/µm² (as estimated from our in vivo data), and then averaged to mimic powder-averaging of magnitude images, which increases the SNR but does not lower the Rician floor. A dataset of 10,000 ground truth combinations was generated in this way.**Dataset 2:**A similar synthetic dataset was produced using bounds derived from the experimental data estimates, [1 - 110] ms for*t*_ex_, [2.5 - 3.5] µm²/ms for*D_i_*, [0.5 - 1.5] µm²/ms for*D_e_*and [0.3 - 0.5] the fraction*f.*For each ground truth, the noise realization followed an SNR that was randomly picked from the SNR distribution of the experimental data. In order to assess the performance of NEXI and NEXI_RM_in the presence of a dot compartment, we built**Dataset 3**in the same way as Dataset 2, but using NEXI_dot_as ground truth with*f_dot_*within [0 - 0.1].

### 2.3 Comparison between NEXI model variants

The four NEXI model variants (Eq. 1-4) were fit to the synthetic and experimental data by Nonlinear Least Squares (NLS) using the L-BFGS-B algorithm and minimize function from the package scipy.optimize (Virtanen et al., 2020), with a tolerance of 1e-14. The bounds specified for the optimization were the same as those described above for the simulations. For the models with a dot compartment, we fitted f_dot_as an additional parameter, with bounds of [0.0001 0.3]. For the models with Rician mean correction, σ was fixed to the noise level estimated in 2.1.3 for experimental data, and to the noise level set in the simulations for synthetic data. To assess the impact of a misestimation of σ in MP-PCA on the performance of NEXI_RM_, σ was also fixed to a value overestimated by 10%, 20%, and 50% of the actual noise level set in the simulations on Dataset 1. The metric used for the optimization was the Mean Square Error (MSE) of the estimated signals against the measured or simulated signals. An initial grid search was applied before the NLS to find an optimal starting point.

#### 2.3.1 Performance in synthetic data

The comparison of the model performance was based on the Median Absolute Error (MedAE) between ground truth and estimation of each model, on the four parameters of interest. This metric was chosen to observe both the real performance of the model and the variance of this performance. The MedAE is more robust to outliers and thus more representative of the performance of the model than Root Mean Square Error (RMSE).

#### 2.3.2 Performance in experimental data

To compare the fit of the four models on our experimental data, one of the criteria used was the corrected Akaike Information Criterion (AICc) (Akaike, 1973). The AICc is a measure used in statistical modeling to assess the goodness of fit of a model while penalizing for its complexity, aiming to balance the trade-off between model accuracy and simplicity.

Furthermore, since both the dot compartment and the Rician noise floor account for the diffusion signal not decaying asymptotically to zero, the dot compartment estimation*f*_dot_in NEXI_dot_was compared to the Rician floor derived from the noise standard deviation in each ROI, estimated using MP-PCA and used as an input to NEXI_RM_.

#### 2.3.3 Repeatability and brain region-specific patterns

Intra-subject versus inter-subject variability was assessed on average GM median ROI estimates obtained by the NEXI_RM_model using Bland-Altman plots (Altman & Bland, 1983).

The spatial distribution of GM microstructure features quantified using NEXI_RM_was also examined using inflated brain surfaces obtained using Connectome Workbench (Marcus et al., 2011) and compared to distribution patterns of neurite density and myelination from the Glasser MRI atlas (Glasser et al., 2022).

## 3 Results

### 3.1 Simulations

**Fig. 2.**
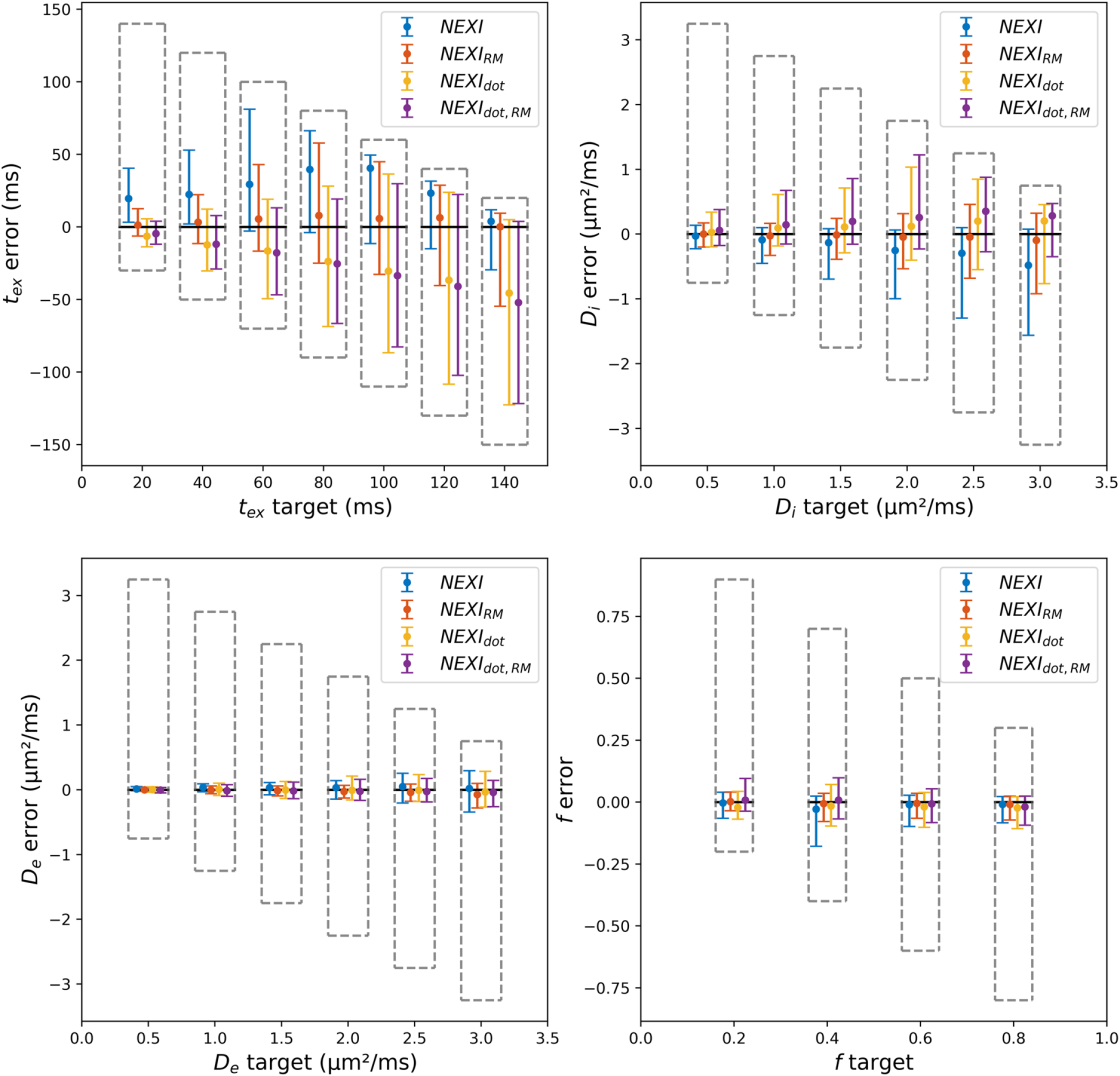
Boxplots (median and interquartile range) of parameter estimates by each of the four model variants on synthetic Dataset 1 with random Rician noise (σ = 0.03). The error is defined as the difference between the estimation and the target value. The upper and lower limits of the grey dashed box represent the maximum and minimum error of the estimator, in the given bin, due to lower and upper bounds in the NLS algorithm.

Given the broad parameter ranges spanned by the synthetic Dataset 1 ground truths, we provide a binned representation of estimation error (Fig. 2). For parameters with the highest estimation uncertainty,*t*_ex_and*D*_i_, the upper and lower bounds on the estimation yielded very asymmetric distributions for bins with ground truth values near those bounds (e.g., for*t*_ex_target ~140 ms or*D*_i_target ~ 3.0 µm^2^/ms).

The neurite fraction*f*and extra-cellular diffusivity*D*_e_estimates benefit from good to excellent accuracy and precision with any model variant. For the two parameters with higher bias and uncertainty (*t*_ex_and*D*_i_): The accuracy on*t*_ex_and*D*_i_was markedly reduced using NEXI, NEXI_dot_and NEXI_dot,RM_as compared to NEXI_RM_, as well as the precision on*t*_ex_using NEXI_dot_and NEXI_dot,RM_.

For a 50% overestimation of σ in Dataset 1, the NEXI_RM_errors are comparable to those of the other models (Table 1andSupplementary Fig. S1). This indicates that some error in the σ estimation from MP-PCA can be tolerated within the NEXI_RM_model. Releasing σ as a free model parameter in NEXI_RM_yielded either similar values to MP-PCA, or a convergence of σ to zero and poorer AICc (data not shown).

**Table 1.** MedAE of NEXI_RM_using the true and overestimated σ, on synthetic Dataset 1 with random Rician noise (true σ = 0.03).

MedAE of NEXI _RM_ using:	*t*_ex_ (ms)	*D*_i_ (µm²/ms)	*D*_e_ (µm²/ms)	*f*
Ground truth σ	22.2	0.393	0.060	0.044
110% σ	22.8	0.389	0.065	0.045
120% σ	24.6	0.389	0.073	0.047
150% σ	31.1	0.413	0.099	0.055

Note the synthetic data spanned broad parameter ranges of ground truths, thus these summary statistics are only partially informative.

Estimation errors on synthetic Dataset 2 (Table 2A) show the NEXI_RM_model yields*t*_ex_estimates with an over 50% lower MedAE compared to all the other model variants. The estimates of neurite fraction*f*and extracellular diffusivity*D*_e_are also substantially improved using the NEXI_RM_model, lowering the MedAE by at least 25% and 40%, respectively. Remarkably, estimation errors on synthetic Dataset 3 (Table 2B) show the NEXI_dot,RM_model yields the lowest errors, closely followed by NEXI_dot_. The errors using NEXI_RM_are double those of NEXI_dot_and NEXI_dot,RM_, which suggests that the Rician mean correction is not able to substitute for the dot compartment.

**Table 2.** MedAE of the different model variants on synthetic dataset generated with NEXI (A.) and NEXI_dot_(B.) with Rician noise. A.

MedAE on NEXI data	*t*_ex_ (ms)	*D*_i_ (µm²/ms)	*D*_e_ (µm²/ms)	*f*
NEXI	28.3	0.63	0.05	0.04
NEXI __RM__	**11.7**	**0.34**	**0.03**	**0.03**
NEXI __dot__	26.1	0.52	0.05	0.07
NEXI __dot,RM__	25.1	0.44	0.06	0.06

**Table:** B.

MedAE on NEXI __dot__ data	*t*_ex_ (ms)	*D*_i_ (µm²/ms)	*D*_e_ (µm²/ms)	*f*	* f __dot__*
NEXI	61.1	1.41	0.13	0.14	-
NEXI __RM__	53.3	1.26	0.11	0.13	-
NEXI __dot__	24.7	0.60	0.05	0.06	0.014
NEXI __dot,RM__	**24.2**	**0.57**	**0.05**	**0.06**	**0.012**

The smallest errors are shown in bold.

Since the synthetic Datasets 1 and 2 were generated assuming a model of two exchanging compartments, it is expected that NEXI_RM_variants perform better than NEXI_dot_variants. However, the simulations underline that failing to account for the Rician floor in the NEXI fit, when Rician noise is present in the data, results in a drastic deterioration of the quality of estimates (NEXI vs NEXI_RM_). They also reveal that the dot compartment fails to mitigate the error due to Rician noise. Introducing a dot compartment in the model when it is not present in the data results in a deterioration of estimates for all other model parameters, in particular for the exchange time (NEXI_dot_and NEXI_dot,RM_vs NEXI_RM_). Conversely, ignoring the dot compartment in the model when it is present in the data results in a deterioration of estimates for NEXI and NEXI_RM_.

### 3.2 Experimental

Based on the DKT parcellation, median values across GM ROIs for each of the model variants are presented inTable 3. The four model variants give very different exchange time estimates. Notably,*t*_ex_estimates are ordered as NEXI > NEXI_RM_> NEXI_dot_. All these estimates are also much longer than 3-5 ms, as reported using NEXI_dot,RM_(though the latter was comparable to NEXI_dot_in the simulations) and previously in*ex vivo*data (Jelescu & Uhl, 2022;Olesen et al., 2022). The extra-neurite diffusivity estimates are comparable across methods. Three of the four models give an intra-neurite diffusivity very close to the upper limit, indicating that the model often hit the bounds, and it may be missing a component to explain experimental data well. The first three methods seem to agree for an average*f*around 0.35 while NEXI_dot,RM_places it higher, at 0.47. In terms of goodness of fit, NEXI_RM_displays the lowest AICc of all models. When comparing between models with and between models without Rician mean correction, NEXI_dot_has a better corrected AICc than NEXI, but the opposite happens when we add the Rician correction, NEXI_RM_outperforms NEXI_dot,RM_.

**Table 3.** Mean estimates and 95% confidence intervals of the median in every ROI of the DKT atlas using NEXI, NEXI_dot_, corrected for rice mean (RM) or not.

	*** t _ex_*** (ms)	*** D _i_*** (µm²/ms)	*** D _e_*** (µm²/ms)	***f***	*** f _dot_***	**AICc**
**NEXI**	**103.9** [100.3, 107.5]	**2.79** [2.71, 2.88]	**0.95 ** [0.94, 0.96]	**0.32** [0.318, 0.325]	**-**	**-139.3** ± 15.0
** NEXI _dot_**	**14.3** [12.2, 16.3]	**3.36 ** [3.32, 3.40]	**1.00** [0.99, 1.01]	**0.36** [0.35, 0.37]	**0.03 ** [0.033, 0.037]	**-140.8** ± 15.6
** NEXI _RM_**	**42.3 ** [40.0, 44.7]	**3.35** [3.32, 3.38]	**0.92 ** [0.91, 0.93]	**0.38 ** [0.379, 0.389]	**-**	**-143.0** ± 16.0
** NEXI _dot, RM_**	**2.90** [2.71, 3.09]	**3.36 ** [3.34, 3.39]	**1.03** [1.01, 1.04]	**0.47 ** [0.47, 0.48]	**0.01 ** [0.009, 0.010]	**-141.6** ± 16.7

Mean estimates are shown in bold.

The last column shows the mean corrected Akaike Information Criterion (AICc) for each model; lower AICc indicates a better fit.

The mean fitted powder-average signal in the whole cortical ribbon by the four model variants is shown inFigure 3. The quality of fit shows that at high b-value and high diffusion time, NEXI performs poorly compared to the other models. However, there is limited agreement between the mean signal and all the models mean fitting curves at high b-value. This is due to the trade-off of fitting the signal across the entire b-value range (Supplementary Fig. S2). Furthermore, the lower quality of the average fit is due to voxels in the cortical ribbon with high AICc, which likely correspond to voxels with substantial partial volume effect, where the model is not performing well.

**Fig. 3.**
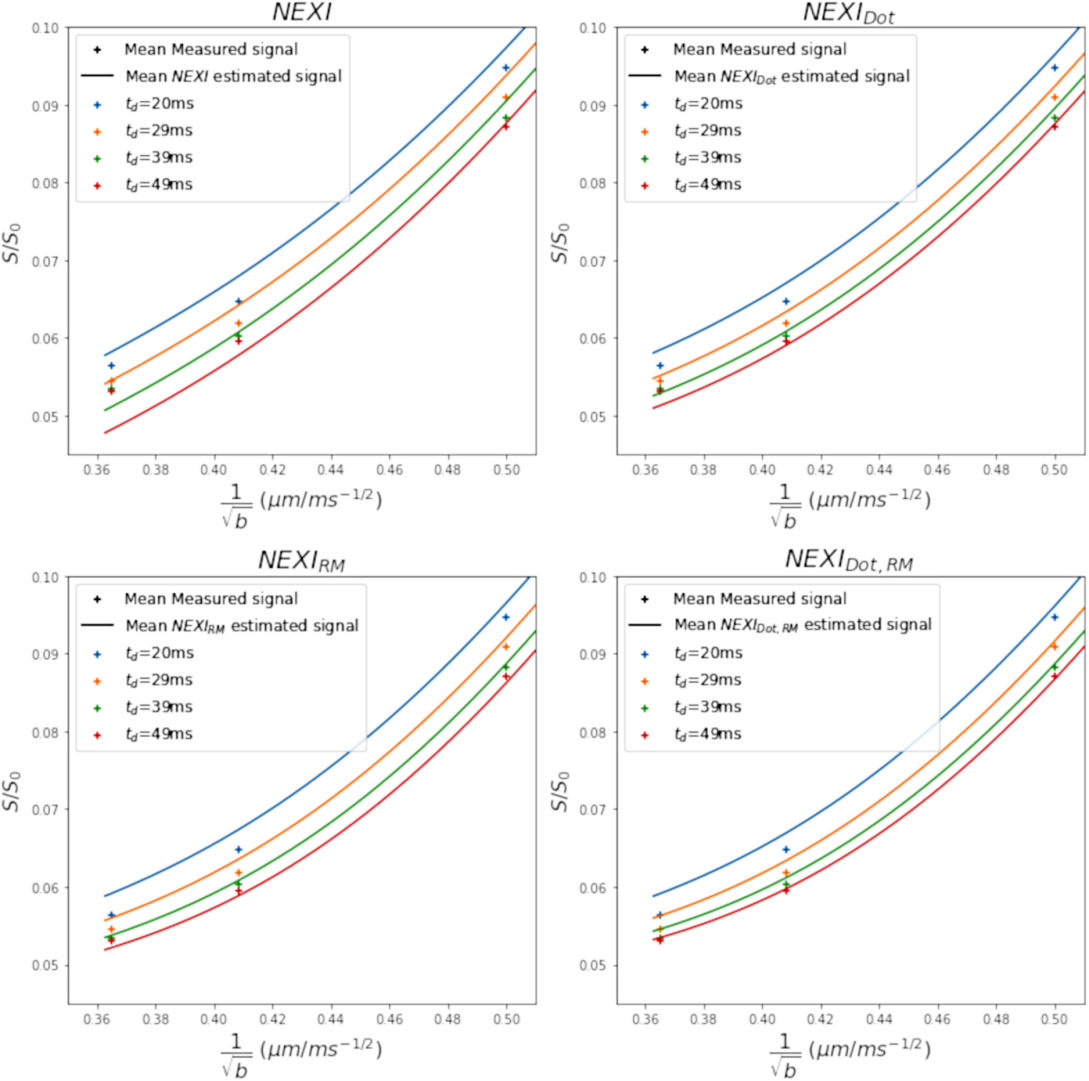
Mean estimated signal in the cortical ribbon by the four NEXI model variants at high b-value (b > 4 ms/µm²) compared to the mean measured signal, represented by plus signs. Each color represents a different diffusion time.

**Fig. 4.**
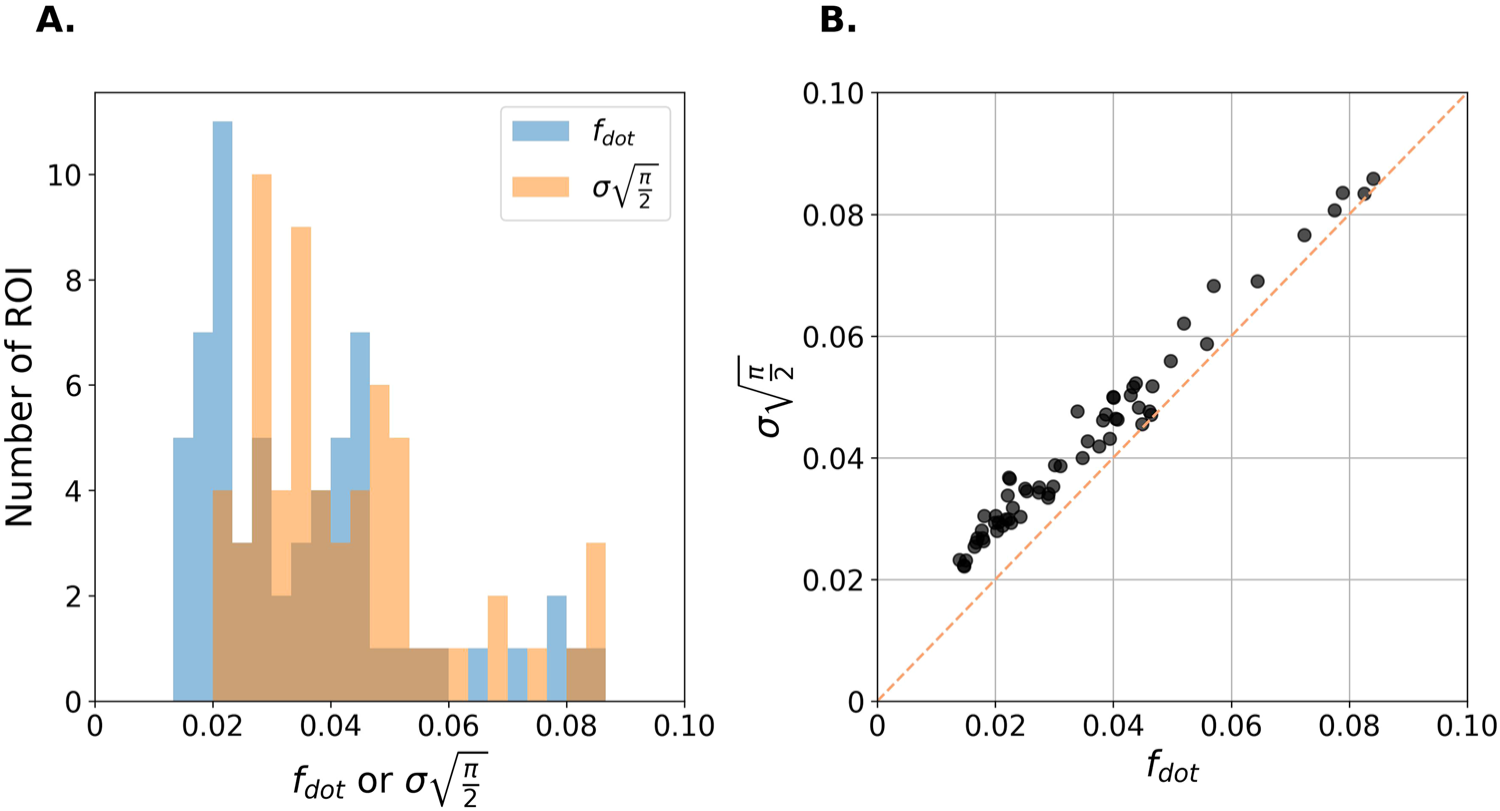
Agreement between f_dot_estimate of NEXI_dot_and the Rician floor expectation value, derived from the noise standard deviation (σ) obtained by denoising small b-values during preprocessing.**(A)**Overlay of f_dot_and σ√(π/2) value distributions across DKT atlas ROIs.**(B)**Correlation between these two quantities. Each point represents the average in an ROI of the DKT atlas.

Furthermore, the dot fraction*f*_dot_estimated using NEXI_dot_was perfectly correlated with the Rician expectation value$\sqrt{\frac{\pi }{2}}\sigma$in each ROI (Fig. 4). The Kolmogorov-Smirnov (KS) test reveals that*f*_dot_andσdistributions are similar (*p*= 0.1967). This suggests that the dot compartment in NEXI_dot_is fitting the Rician floor with a systematic offset, casting doubt on an actual dot compartment being relevant for cortical GM in vivo, in agreement with (Tax et al., 2020) and that the NEXI_RM_model should therefore be preferred.

**Fig. 5.**
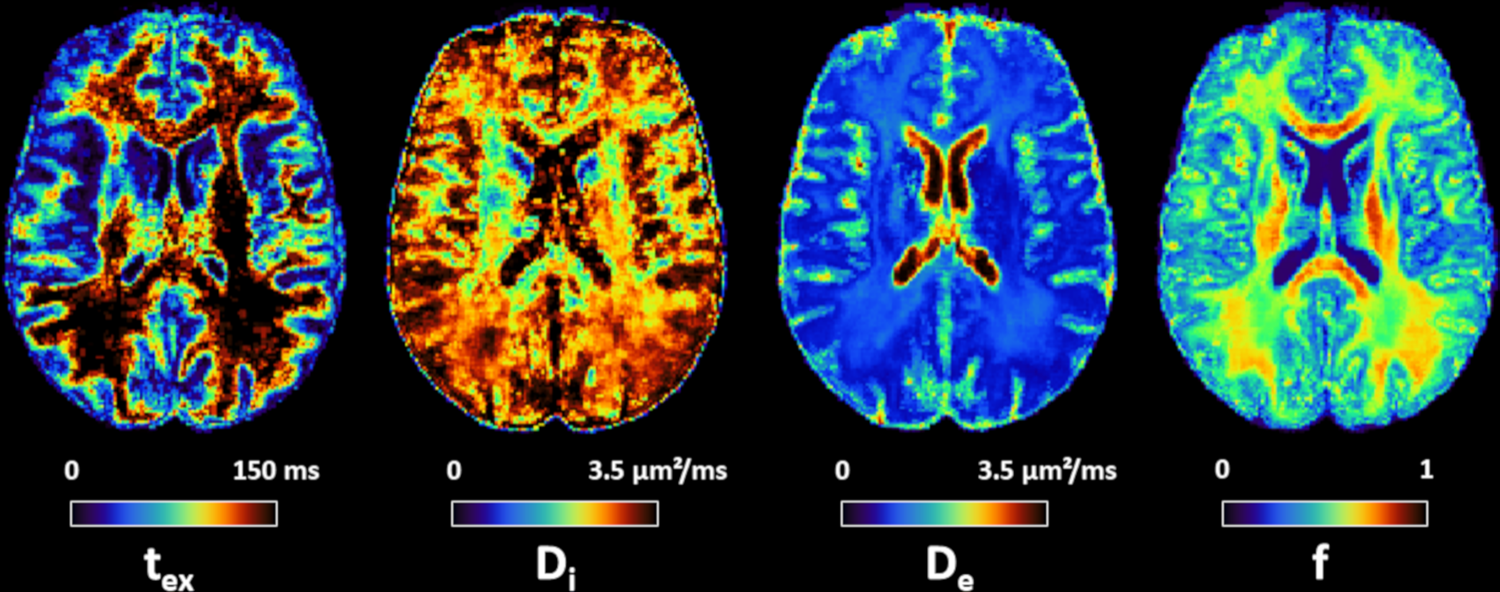
Axial slice of NEXI_RM_parametric maps, averaged across sessions and subjects (N = 7).*t*_ex_and*D*_e_are consistent throughout the cortex, but*t*_ex_is presumably longer in the WM and cannot be reliably estimated using available diffusion times.*f*displays the expected anatomical pattern in white versus gray matter.*D*_i_shows large variability across voxels, while hitting its upper bound frequently.

Based on this model variant comparison which favors the use of NEXI_RM_in vivo, we report NEXI gray matter microstructure estimates in the human brain (Fig. 5). Using the NEXI_RM_implementation, quantitative maps show, as expected,*t*_ex_estimates in the range 20 – 50 ms in the cortex, and much longer in the white matter, where the diffusion time range does not allow a reliable estimation. The*D_e_*map shows lower values in the cortex compared to sub-cortical WM. This aligns with the idea that the high cellular abundance and random neurite orientations in GM slow down extra-cellular diffusion. In contrast, WM experiences less hindrance to diffusion, especially along axons. The*D*_e_contrast may also be consistent with the soma compartment being absorbed into the extra-cellular compartment in NEXI, thereby reducing its apparent diffusion in GM by the inclusion of restricted components. The neurite density fraction map reveals expected WM/GM contrast, with much higher fraction in WM; the cortical neurite fraction is estimated at ~40%. It should be noted that NEXI is not designed for WM, where the assumption of randomly oriented sticks and isotropic extra-neurite diffusivity is not expected to hold. This could have affected estimates in single-fiber WM population voxels versus crossing fiber WM areas, for example.

**Fig. 6.**
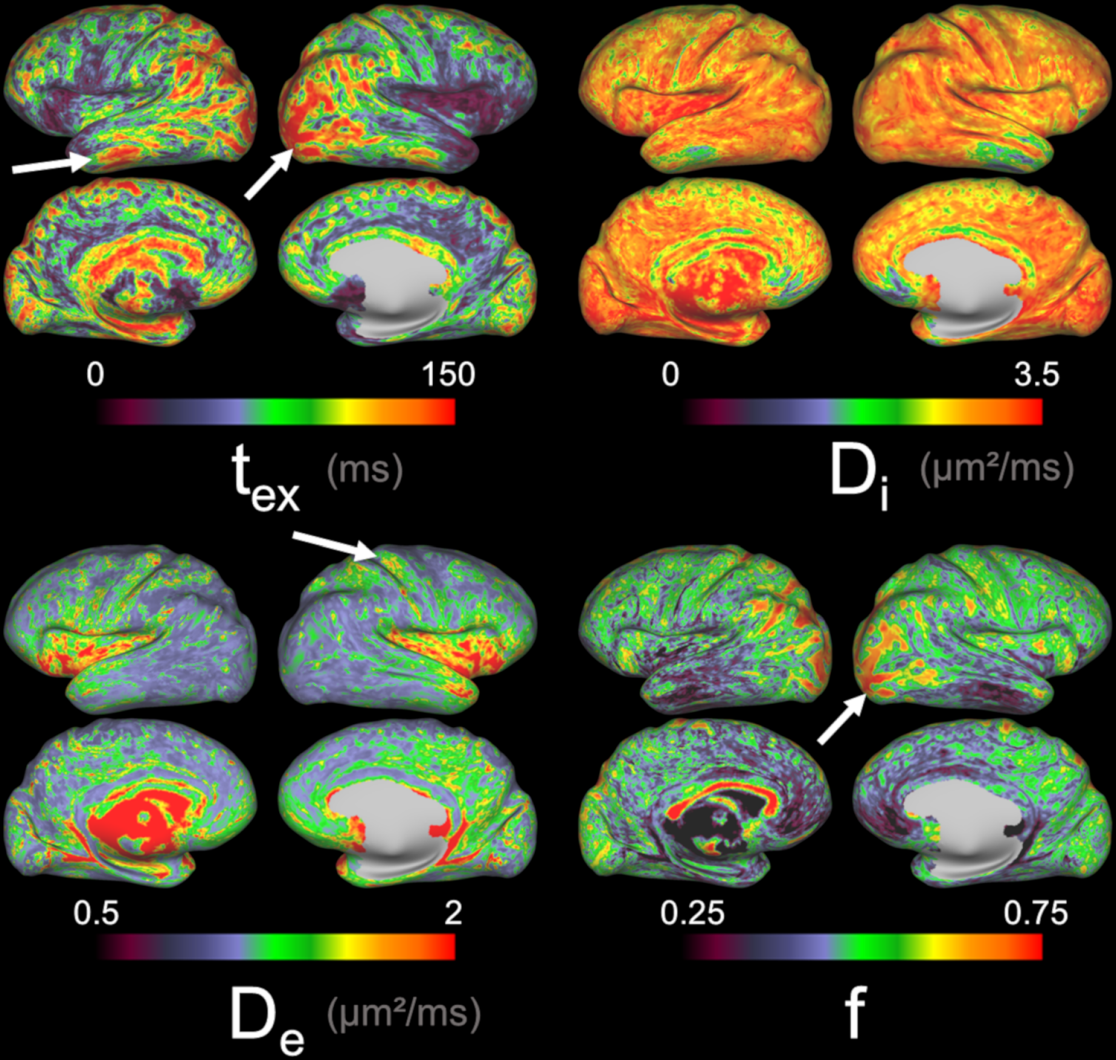
Projection onto cortical surface of NEXI_RM_maps averaged across subjects and sessions. We find some of the expected pattern of a larger fraction of neurites in the occipital lobe (white arrow). This pattern can also be seen in the exchange time, which is also longer in the temporal lobe (white arrows). A higher extracellular diffusivity is also observed in the somatosensory cortex (white arrow).

These parametric maps, averaged within each DKT ROI, projected onto a study-average inflated cortical surface and averaged at the cortical thickness level voxel-wise after a multivariate template registration (Fig. 6), reveal remarkable patterns across the healthy human brain. First, there is an expected level of symmetry between left and right hemispheres, although their estimates are completely independent, which suggests that spatial patterns are not casual.

Figure 6shows that the longest exchange time was found in the occipital lobe, in the posterior part of the parietal lobe, and in the ventral parts of the temporal lobe, possibly indicating correlation with cortical myelination.*D_i_*estimates reach the upper bound in most of the regions of interest, limiting interpretation. However, a decrease in*D_i_*is observed in the rostral and ventral parts of the temporal lobe.*D_e_*revealed spatial patterns of faster extra-cellular diffusivity along the somatosensory cortex, as opposed to the occipital lobe and caudal part of the temporal lobe which have the slowest*D_e_*. In the insula,*D_e_*is also considerably faster; however, the level of partial volume effects might be higher, biasing the estimates upwards. As suggested above,*D_e_*is likely impacted by cellular density (extra-cellular tortuosity and high soma density) which reduces its estimate, or by fiber alignment that increases its estimate. Lastly, the neurite fraction*f*follows a pattern of highest density in the occipital lobe and in the caudal part of the parietal lobe, comparable to*t_ex_*pattern possibly linked with myelination, but with moderate to lower densities in the ventral part of the temporal lobe.Supplementary Figure S3presents a comprehensive depiction of these results, showcasing the parametric medians per region of interest.

#### 3.2.1 Agreement with time-dependent diffusion and kurtosis

**Fig. 7.**
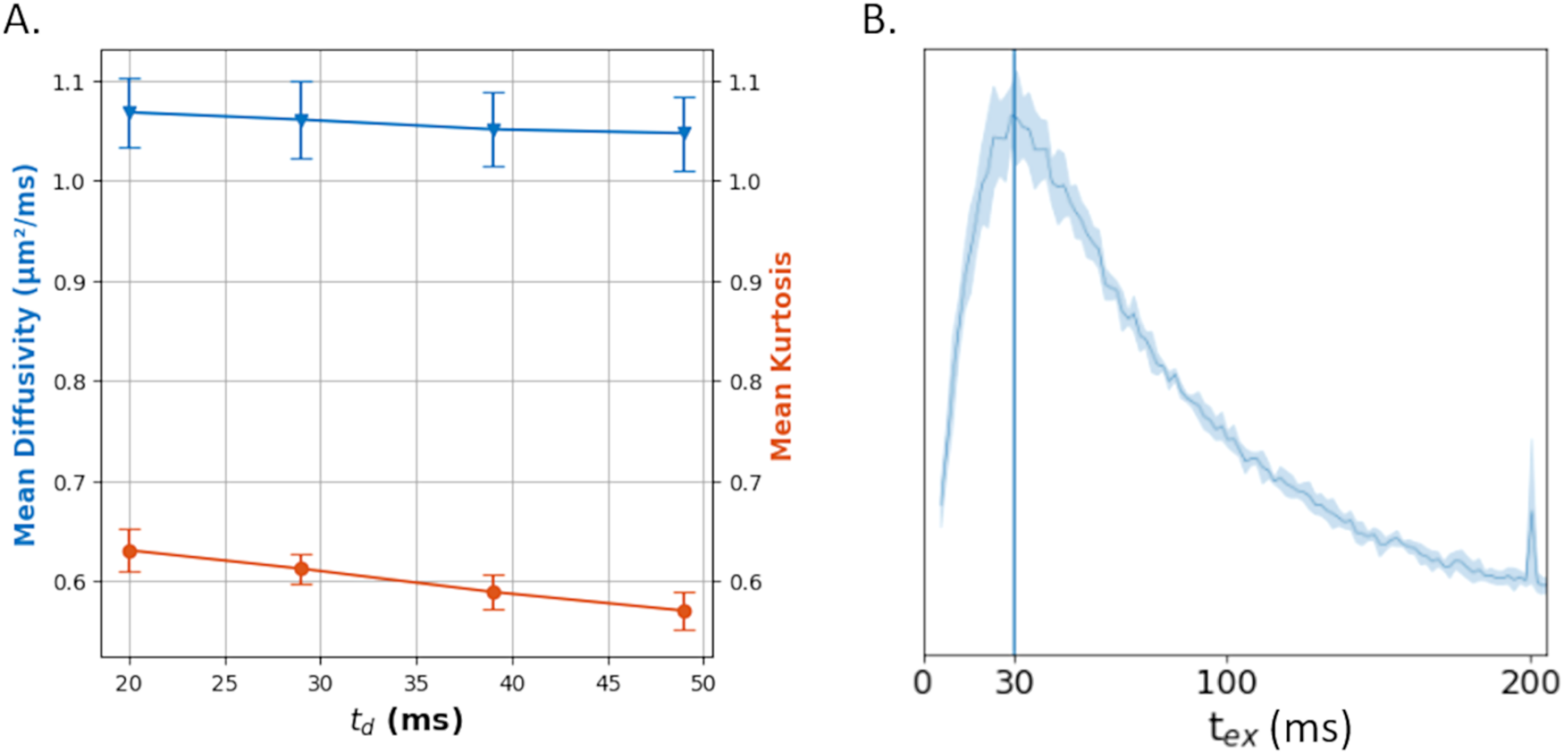
**(A)**Time-dependent Mean Diffusivity and Mean Kurtosis in the cortex, averaged over voxels in the cortical ribbon and across the seven datasets (subjects and sessions).**(B)**Distribution of$t_{ex}^{K\left(t\right)}$values estimated voxel wise across the cortex, averaged across subjects (first session).

Mean Diffusivity was almost independent of the diffusion time, with a weak yet measurable slope of$-7.5\text{x}10^{-4}{\text{ µm}}^{2}/{\text{ms}}^{2}$(*p*= 0.01) (Fig. 7A). This diffusion time-dependence, albeit weak, potentially calls into question the assumption of Gaussian compartments in our models. This would be consistent with a minor degree of structural disorder, encapsulating the subtle heterogeneities within the compartments (Lee et al., 2020), potentially impacting the model's precision. Mean Kurtosis decreased more markedly with time, which is consistent with previous studies (Jelescu et al., 2022;Lee et al., 2020). We find good agreement between$t_{ex}^{KM}$obtained from MK(t) analysis and the one obtained from the NEXI_RM_fit. This agreement is expected as MK(t) in Eq(5)uses low b-value data that are less affected by Rician floor than the full NEXI model (Eqs.(1)and(3)).

##### 3.2.2 Inter- versus intra-subject variability

**Fig. 8.**
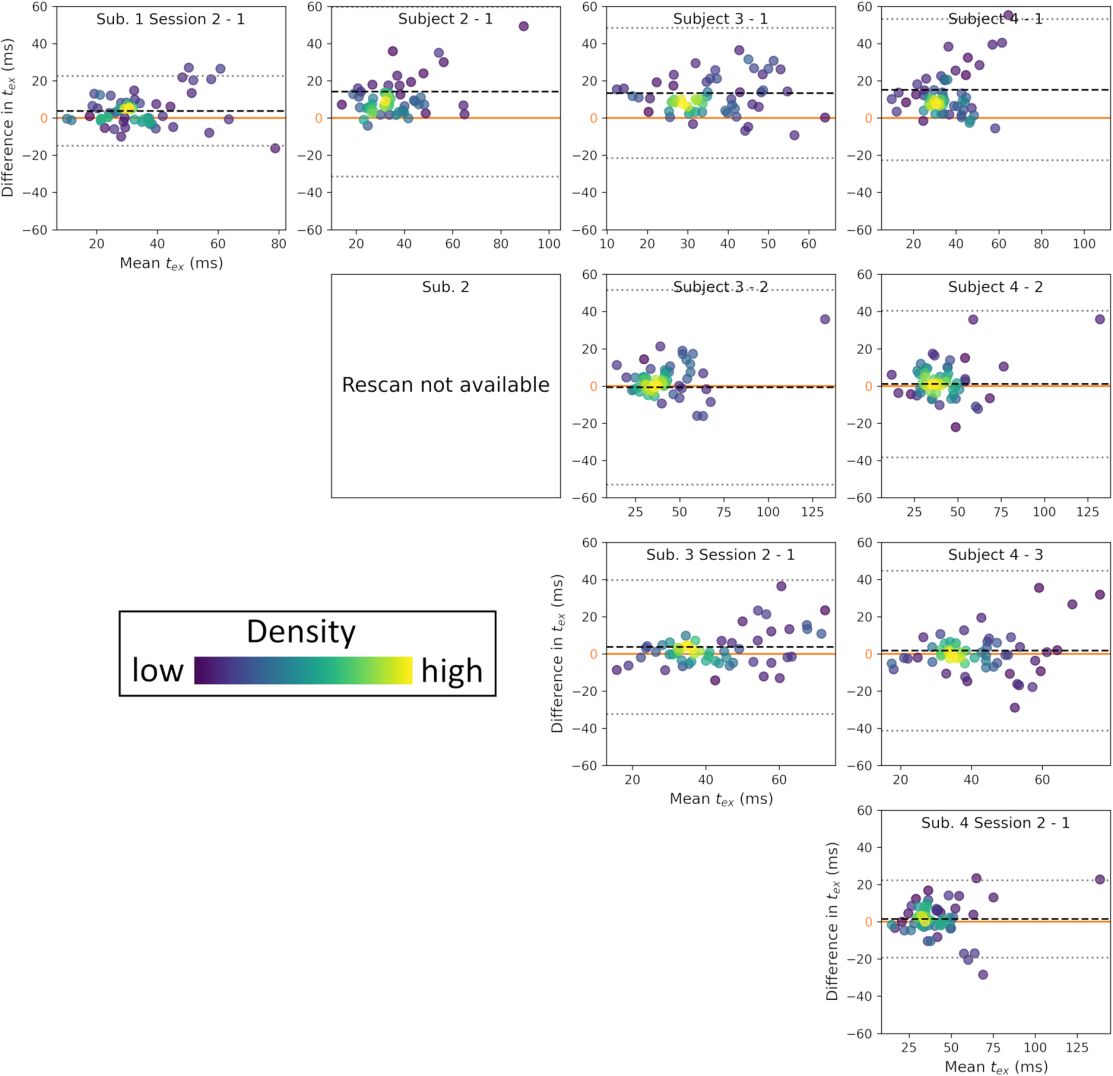
Bland-Altman plots of the t_ex_estimations from NEXI_RM_model. Each row and column refer to the same subject. On the diagonal, the two sessions of each subject are compared. In the upper triangle, the results of the first session of each subject are compared to those of another subject. The colors reflect the density of points on the plot, each of them representing the mean value in a given DKT ROI.

To assess intra-subject variability, we compared the first and second sessions of the three subjects who were scanned twice. To assess inter-subject variability, we compared the first session of the four subjects between them. Below, we compared NEXI_RM_results (Figs. 8and9); for the other models, the plots are provided inSupplementary Figure S4.

**Fig. 9.**
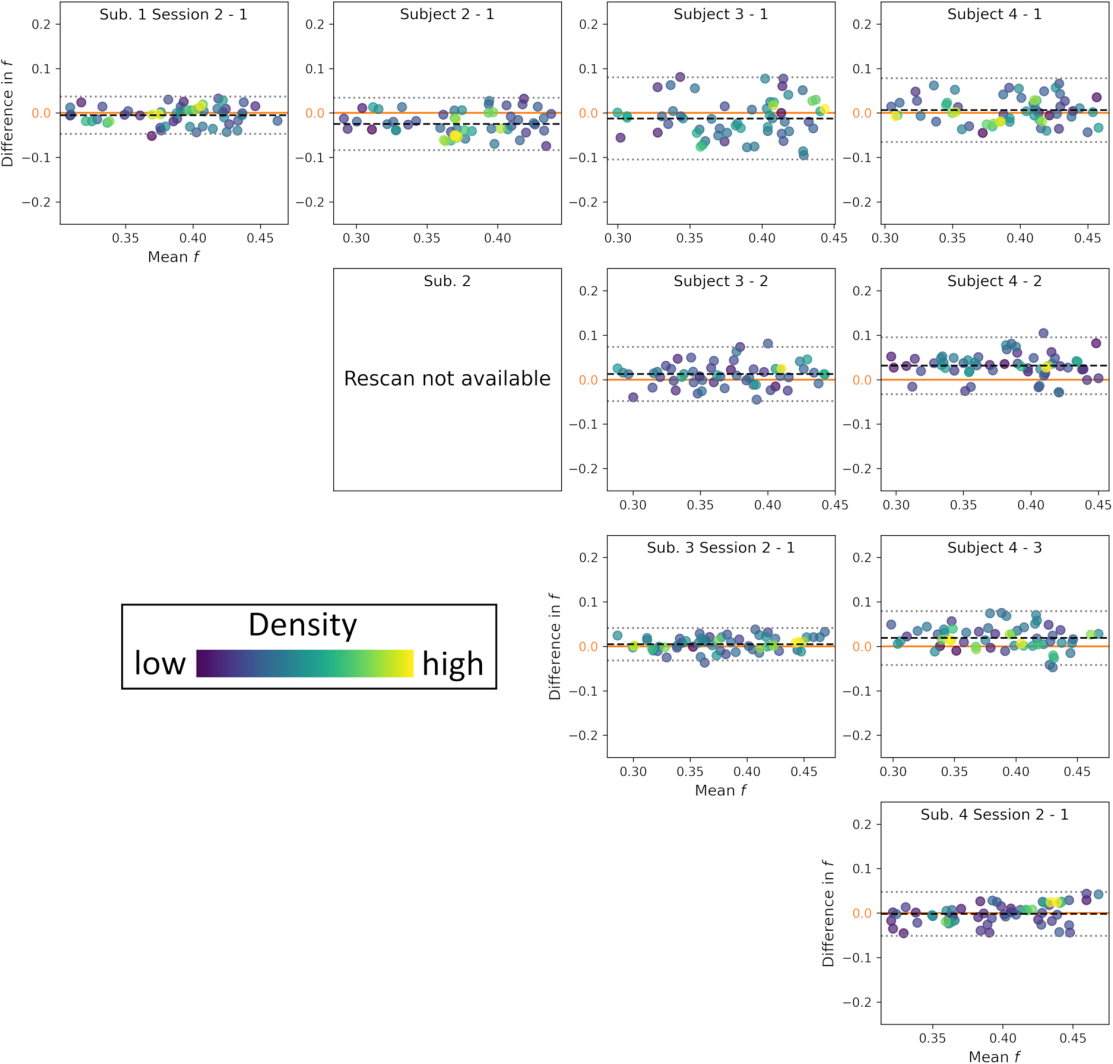
Bland-Altman plots of the f estimations from NEXI_RM_model. Each row and column refers to the same subject. On the diagonal, the two sessions of each subject are compared. In the upper triangle, the results of the first session of each subject are compared to those of another subject. The colors reflect the density of points on the plot, each of them representing the value in a given DKT ROI.

The difference in median*t*_ex_over each ROI between different sessions is approximately 3.0 ms, while the difference in*t*_ex_across subjects is more than 2.5 times larger, at 7.70 ms (Fig. 8). It is also noteworthy that the*t*_ex_do not display a broad range across the ROIs, with most values concentrated between 40 – 60 ms.

In terms of neurite fraction*f*, the mean difference increases from 0.0040 for the inter-session comparisons to 0.01770 for the inter-subject comparisons (Fig. 9), that is, intra-subject variability is over four times larger than scan-rescan variability, a difference even more pronounced than for*t*_ex._The variance is also higher in the inter-subject versus intra-subject comparisons. Unlike the exchange time*t*_ex_, neurite fraction values cover a broader range across DKT ROIs, showing brain regional specificity of this parameter.

This suggests that NEXI_RM_estimates are sufficiently reproducible to retain sensitivity to inter-subject differences.

For comparison, the Bland-Altman plots for*t*_ex_and*f*of the other models can be found inSupplementary Figure S4. Additionally, the Bland-Altman plots for the two diffusivities of NEXI_RM_are available inSupplementary Figure S5.

## 4 Discussion

In this study, we compared different variants of the NEXI model in order to quantify microstructure features in the human cortex. We thus compared NEXI estimates, implemented as a two-compartment model with exchange as inJelescu et al. (2022), to those from its three-compartment variant NEXI_dot_, also accounting for a dot compartment as proposed inOlesen et al. (2022)for ex vivo data, as well as two new versions that correct for the Rician bias in the signal at high b-values: NEXI_RM_and NEXI_dot,RM_. By examining these four model variants, the goal was to investigate the pertinence of a dot compartment to model human cortical gray matter, similar to the one identified in the cerebellum (Tax et al., 2020), and to study the effect of the Rician noise correction on these two models, given the lower SNR of clinical dMRI data as compared with preclinical data.

In the case where the ground truth is a two-compartment model with exchange and the standard deviation of the noise is known, the simulation results clearly show that the NEXI_RM_model is to be preferred against the other models and that the dot compartment is not able to substitute the Rician noise correction efficiently. Similarly, adding both a dot compartment and a Rician noise correction seems to disturb the model in the estimation of the main parameters, likely by the addition of an unnecessary free parameter (*f*_dot_). The bias in NEXI estimates when the Rician floor is not accounted for is also very marked, although this bias is expected to be dependent on the SNR of the data. Simulations show that the performance of NEXI_RM_is equivalent to the performance of the other models in the case where the estimation of the noise level input into the Rician mean correction is overestimated by 50%.

While the RM correction is clearly beneficial, the performance of the three-compartment NEXI_dot_model on synthetic data generated using the two-compartment NEXI model is challenging to interpret. On the one hand, it is obvious that a non-zero dot compartment will be estimated, even when it is absent in the ground truth. On the other hand, if a dot compartment is present in the ground truth, NEXI_RM_is not able to account for that as it uses the realistic σ value for the Rician floor as would be typically obtained from MP-PCA denoising. It is important to underline that the existence of the dot compartment in healthy*in vivo*cerebrum tissue is not highly supported by histological evidence or previous experiments using spherical diffusion tensor encoding (Tax et al., 2020). Furthermore, NEXI_RM_and NEXI_dot_fits on experimental data show that NEXI_dot_essentially captures the Rician noise floor as a dot compartment, rather than the latter having a biological relevance as a compartment of its own. The slight but systematic lower level of the*f*_dot_estimate compared to the Rician floor$\sigma \sqrt{\frac{\pi }{2}}$could be explained by the fact that the Rician correction is adaptive, mainly changing the signal magnitude at low SNR (high b-values) while the dot compartment acts by design as an offset to the signal across the entire b-value range. Thus, the*f*_dot_estimate is likely lower than the Rician floor as a compromise in fitting the signal well at both low and high b-values, in an MSE sense.

It is also noteworthy that NEXI_RM_was the model with the lowest AICc, while a potential error on σ could have further reduced the performance of the NEXI_RM_fit, as also shown in the simulations of an overestimated σ. When comparing between models with or without Rician mean correction, our results show that the Rician correction is always beneficial, whether a dot compartment is modeled or not. On top of this result, the better AICc of the models with Rician Mean correction shows that the a priori input σ value provides a more precise fit.

Overall, our results on both synthetic and experimental data therefore indicate that the NEXI_RM_model, that is NEXI corrected for Rician noise, should be preferred for in vivo human cortex. It is noteworthy that the dot compartment may nonetheless be relevant as a biological compartment of its own in ex vivo data (Olesen et al., 2022). Furthermore, a soma compartment may be needed to better account for the signal decay at high b-values, although a model accounting for both soma and exchange (such as SANDIX) would likely require more datapoints and high SNR to yield reliable fit estimates, as discussed in the limitations paragraph.

The NEXI_RM_average estimate of*t_ex_*in the human cortical ribbon is 42 ms, versus 104 ms for NEXI. The former matches well with the average$t_{ex}^{K\left(t\right)}$of 30 ms from the time-dependent kurtosis analysis, which is expected since$t_{ex}^{K\left(t\right)}$is derived from data with b ≤ 2.5 ms/µm^2^which have higher SNR and are thus less impacted by the Rician floor. It is remarkable how different*t*_ex_estimates are across the four model variants, with the inclusion of a dot compartment systematically reducing*t*_ex_. While the ground truth*in vivo*is not known, simulations support the experimental ordering in*t*_ex_estimates across models, with NEXI yielding the highest (and over-estimating*t*_ex_in simulations), followed by NEXI_RM_(with best accuracy in simulations), and finally NEXI_dot_and NEXI_dot,RM_. Indeed, fitting an experimental signal that does not effectively decay to zero using a model that predicts a signal decay to zero at high b-values (as NEXI) will result in an overestimated exchange time*t_ex_*. When the Rician floor is introduced in the model, a more accurate*t_ex_*can be estimated. The non-adaptive offset in NEXI_dot_results on the contrary in an underestimated*t_ex_*vs NEXI_RM_. The difference between NEXI_dot_and NEXI_dot,RM_is more pronounced in experimental data than in simulations, which could be attributed to partial volume effects or other tissue compartments not accounted for in the models, and that were absent in the simulations. Overall, these discrepancies reinforce the need to make informed decisions when selecting the model, as these decisions have a dramatic impact on ensuing exchange time estimates.

The estimation of other parameters is less variable across models. Extra-neurite diffusivity,*D*_e_, is 0.9 – 1 µm^2^/ms, slightly higher than that reported in rats using NEXI (Jelescu et al., 2022). The neurite fraction,*f*, is ~0.3 – 0.4, also consistent with what has been reported in rats using NEXI (Jelescu et al., 2022). However, the obtained neurite volume fraction from histology, approximately 60% in the rat cortex (Braitenberg & Schüz, 1998;Chklovskii et al., 2002;Ikari & Hayashi, 1981), is much larger. This discrepancy may be due to a relaxation bias. In WM, T_2_is likely shorter in the extra-axonal than in the intra-axonal space. In contrast, GM shows the opposite pattern, with less extracellular myelin and factors like cytoskeleton and neurofilaments possibly shortening intra-neurite T_2_. Shorter intracellular T_2_relaxation times are illustrated in MR microscopy of human and porcine neurons (Flint et al., 2012). Such differences in T_2_could result in an underestimation of the compartment’s volume fraction, or to faster exchange processes not captured by*t*_ex_, that would also result in an underestimation of the restricted stick population.

However, for all models, the intra-neurite diffusivity measure,*D*_i_, is unrealistically high, even above water diffusion coefficient at the body temperature of 3 µm²/ms, and often hits the upper bound implemented in the NLS algorithm. Intra-neurite or intra-axonal diffusivity is notoriously challenging to estimate, particularly in the presence of noise (Howard et al., 2022;Jelescu et al., 2016;Palombo et al., 2020). One possibility is that larger b-values would be required to estimate*D_i_*in the gray matter (as in the preclinical NEXI implementation (Jelescu et al., 2022) or in the WM Standard Model (Howard et al., 2022)), or a combination of linear diffusion encoding with b-tensor encoding and/or*T_2_*relaxometry, as was the case in white matter (Dhital et al., 2019;Lampinen et al., 2020). Alternatively, working with real-valued data instead of magnitude data could help eliminate Rician bias and boost the SNR, thereby improving the*D*_i_estimates (Howard et al., 2022). The simulations also show*D*_i_to display the largest error estimation relative to its range of possible values. It is also possible that a Partial Volume effect (PVE) takes place, where gray matter, white matter, and CSF are captured in each voxel in varying proportions. This would make the model less suitable for our experimental data, pushing*D*_i_estimates towards unphysical values; the issue of PVE is discussed further below.

Based on the few recent works on gray matter exchange models, there seem to be dramatic differences in cortical gray matter microstructure features between*in vivo*and*ex vivo*tissue. Reported exchange times*ex vivo*are much shorter than in vivo, 3 – 14 ms irrespective of the inclusion or not of a dot compartment, neurite fractions are much higher 0.7 – 0.8, closer to their histological estimates (also based on ex vivo tissue) (Hertanu et al., 2023;Jelescu & Uhl, 2022;Olesen et al., 2022), contributions from structural disorder are more pronounced (Jelescu & Uhl, 2022), and*D*_i_is reduced within biologically plausible ranges (Hertanu et al., 2023;Jelescu & Uhl, 2022). However, other groups have reported very short exchange times (3 – 10 ms) also in perfused viable rat pup spinal cord (Williamson et al., 2023,^2019^), and even in human cortex in vivo (Lee et al., 2022), which would however translate into higher membrane permeability than ever reported for human neurons and astrocytes, as previously discussed (Boss et al., 2013;Jelescu et al., 2022). Similarly, previous works have put forward that structural disorder dominates over exchange in human cortex (Lee et al., 2020), considering detectable time-dependent diffusion in some ROIs. Here, we also report weak yet detectable time-dependent diffusion when averaging across all voxels in the cortical ribbon and across subjects, with a significant negative slope. Although this time-dependence could challenge the assumption of barrier-limited exchange between two Gaussian compartments underlying the Kärger model and thereby NEXI, this contribution seems limited as compared to the exchange that drives a pronounced time-dependent kurtosis. Time-dependent diffusion in the human cortex may also result from PVE with subcortical WM, as it was previously unambiguously reported in human WM (Fieremans et al., 2016), but not in the rat cortex in vivo where PVE with WM could be excluded (Jelescu et al., 2022).

We also report NEXI parameter distributions across the surface of the human brain. While the maps of neurite fraction and exchange time do not fully align with the expected cortical myelin density mapping (Ali et al., 2022;Van Essen et al., 2018), brain regional differences are still in line with known variation in cell density and myelination across the cortex. Overall, longer exchange times, higher neurite densities, and faster extracellular diffusivity (suggesting a more coherent alignment of neuronal processes) were found in motor, somatosensory, and visual areas. It should also be underlined that biophysical models of water diffusion do not provide cell-type specific information, and astrocyte distributions across the cortex may also impact the NEXI maps in terms of “neurite density” (which rather mirrors cell process density) and exchange time (assuming astrocytes may be more permeable than neurons due to the presence of aquaporin-4 channels (Boss et al., 2013;Gleiser et al., 2016;Halnes et al., 2013)). Variations in neurite orientation coherence between regions may also challenge the assumption of isotropic extra-neurite diffusivity and may bias the region-specific estimates based on this.

Finally, NEXI_RM_estimates display good scan-rescan repeatability while retaining sensitivity to inter-subject differences. These results are promising from the perspective of further clinical translation of NEXI, and its application to larger populations of healthy subjects and patients. The NEXI implementation on the Connectom scanner is a steppingstone between preclinical MRI systems and widespread clinical MRI systems. The advent of new human scanners featuring gradient amplitudes of 200 mT/m, such as the Cima.X (Siemens Healthineers) or the MAGNUS (GE Healthcare) (Foo et al., 2020), suggests that the next generation of MRI scanners will increasingly resemble the scanner used in this study, in terms of gradient amplitude, thereby expanding its scope. The potential of NEXI_RM_to estimate cortical microstructure features on a clinical scanner (Uhl et al., 2023) will be strengthened by the results of the present study, as it highlights the importance of correcting for Rician noise in the NEXI model to obtain accurate estimates of microstructure parameters in the human cortex. The Rician mean correction is expected to have even more influence on clinical data with lower SNR (due to the longer TE driven by weaker gradients up to 80 mT/m). The progress in hardware technology, exemplified by advancements like Connectom 2.0 (Huang et al., 2021), as well as the aforementioned new scanners, also holds significant promise for advancing the validation of reproducibility in this study at higher SNR and facilitating future clinical translation.

Our study has some limitations that should be noted. First, this study was a proof of principle, for which we sampled four participants. Future studies with larger sample sizes, possibly including patients, are warranted. Second, several trends suggest that the NEXI_RM_model, though more appropriate than the other three variants, may be incomplete to fully characterize cortical GM signal behavior in (q,t) space. The weak decay of D(t) may indicate that the model assumption of Gaussian compartments does not hold entirely; this should be further investigated on a larger cohort with a broader range of diffusion times. However, accounting for structural disorder explicitly in a biophysical model in combination with exchange is still work in progress for the community (Burcaw et al., 2015;Novikov et al., 2023,^2014^). Furthermore, the soma compartment was neglected from the model, in light of the more pronounced effect of exchange over restriction signified by decreasing signal with increasing diffusion time (Jelescu et al., 2022;Olesen et al., 2022), but should represent a priority for future work. Indeed, quantifying soma at short diffusion times using SANDI has demonstrated value (Palombo et al., 2020) but is also challenging from the perspective of model degeneracy when combined with an exchange model as in SANDIX (Olesen et al., 2022). Recent approaches using different gradient waveforms have been proposed to separate the contributions of exchange (permeability) and restriction (soma) (Chakwizira et al., 2023) but led to much longer exchange time estimates than with NEXI, rather in line with previous literature using FEXI (Lampinen et al., 2017) which lacks specificity to biologically-relevant compartments. Structural disorder has also not been considered in this approach. Residual effects of Rician noise may compromise the intra-neurite diffusivity estimate, which may benefit from working with real-valued vs magnitude data.

Finally, some open choices have been made concerning the algorithms used in our preprocessing and processing pipelines. We applied MP-PCA denoising in the context of parallel image acquisition and spatially correlated noise. However, the AdaptiveCombine reconstruction algorithm effectively preserves noise properties, mitigating issues with correlated noise. The inspection of residuals revealed no anatomical structure. We also applied Gibbs ringing correction using an algorithm not well suited for partial Fourier data. However, the resulting corrected images did not reveal dramatic residual ringing. Given the minimal effect of these steps on our data, we maintain confidence in our preprocessing approach. However, to further enhance our methodology, future studies will incorporate advanced techniques like NORDIC (Moeller et al., 2021) or Efficient PCA (Henriques et al., 2023) for denoising, and adopt a specialized framework for Gibbs ringing correction suggested for partial Fourier data (Lee et al., 2021).

The inspection of denoising residuals also revealed, as expected, weaker denoising at the edge of the brain mask, where the denoising kernel is partially populated. This translates into an underestimation of noise levels at the edges of the mask, potentially bringing the model estimates with and without Rician noise correction closer together. The impact is somewhat mitigated by the higher SNR in brain areas which are in close proximity to the receiver coils compared to, for example, the midbrain. We believe this issue does not significantly affect our results, as all analyses were performed within the cortical ribbon, uniformly influenced by the edge effect.

We also acknowledge the interest in using the Rician Distribution Maximum Likelihood Estimation (Sijbers et al., 1998) for NLS fitting instead of correcting our models for the Rician Mean. However, its high computational demands and incompatibility with our current efficient loss function approach, accelerated by its Jacobian, led us to not employ it in our experiments and simulations. The analytical Jacobian further makes the fit convergence more stable, by limiting the impact of noise on the fitting landscape. This choice was guided by the need for computational efficiency, considering the vast range of parameter combinations we analyzed. Exploring the comparison of these fitting methods remains a potential area for future research.

One of the advantages of the NEXI model is that it can be implemented on clinical scanners (Uhl et al., 2023) and thus enables studies in large cohorts of both healthy and patient populations. Future research will focus on its optimization on a clinical scanner with more moderate gradient set of 80 mT/m, although the availability of clinical scanners with 200 mT/m gradients can only ease the clinical translation of NEXI. Optimization avenues include accounting for the actual gradient pulse duration (as the narrow pulse approximation may not hold, as implemented inOlesen et al. (2022)), trading magnitude data for real-valued data, trading NLS for a multi-layer perceptron fit, and using explainable AI to optimize the clinical NEXI acquisition protocol within scanner hardware limits (Uhl et al., 2023). The main goal is to reduce both the acquisition time, and the estimation error on the two most challenging parameters, namely D_i_and*t*_ex_. The development of a framework that enables joint estimation of soma and neurite permeability is also high priority.

## 5 Conclusion

We reported the first comprehensive study of NEXI model parameter estimates in the human cortex in vivo. Our findings indicate that the addition of a dot compartment to the NEXI model is not necessary and that correcting the Rician floor in the fit is a more appropriate approach to account for its effects, given that the estimated dot compartment correlated very strongly with the noise floor estimated independently from MP-PCA denoising on low b-value data. The estimated exchange time, neurite fraction, and compartment diffusivities are consistent with previous studies conducted in the rat cortex in vivo, as well as with the exchange time estimate from time-dependent kurtosis. Notably, we observed that the exchange time is on the order of 30 – 40 ms, an intermediate value as compared to other similar studies but that signifies exchange cannot be neglected in the human GM at clinical diffusion times. These estimates displayed good scan-rescan repeatability, while preserving sensitivity to variations among subjects. However, the parameters*D*_i_and*t*_ex_were the most challenging to estimate, and future efforts will focus on possible improvements.

## Data and Code Availability

The code used in this study is available onhttps://github.com/Mic-map/nexi. The data used in this study are available upon request after signing a formal data sharing agreement and providing approval from the requesting researcher’s local ethics committee.

## Author Contributions

Conceptualization: I.J., M.P.; Data curation: Q.U., M.M.; Formal analysis: Q.U., T.P.; Funding acquisition: I.J., M.P., and D.K.J.; Investigation: M.M., M.P., and I.J.; Methodology: I.J., Q.U., T.P., M.P., and D.K.J.; Supervision: I.J.; Visualization: Q.U., T.P.; Writing—original draft: Q.U., I.J.; Writing—review & editing: I.J., M.P., D.K.J., M.M., and T.P.

## Funding

Q.U., T.P., and I.J. are supported by SNSF Eccellenza grant PCEFP2_194260. M.P. is supported by UKRI Future Leaders Fellowship MR/T020296/2. The data were acquired at the UK National Facility for In Vivo MR Imaging of Human Tissue Microstructure funded by the EPSRC (grant EP/M029778/1), and The Wolfson Foundation. The work is supported in part by a Wellcome Trust Investigator Award (096646/Z/11/Z) and Wellcome Trust Strategic Award (104943/Z/14/Z).

## Declaration of Competing Interest

The authors declare no competing interest. For the purpose of open access, the author has applied a CC BY public copyright license to any Author Accepted Manuscript version arising from this submission.

## Supplementary Materials

Supplementary material for this article is available with the online version here:https://doi.org/10.1162/imag_a_00104.

## Supplementary Material

Supplementary Material
