# Supplementary material for: Quantifying human gray matter microstructure using neurite exchange imaging (NEXI) and 300 mT/m gradients
Source: Imaging Neurosci (Camb). 2024 Mar 6;2:imag-2-00104. doi: 10.1162/imag_a_00104 (PMC12247613; doi:10.1162/imag_a_00104)
Supplement: Supplementary Material [file imag_a_00104-supp.pdf]

Quantifying human gray matter microstructure using Neurite Exchange Imaging (NEXI) and 300 mT/m gradients

Quentin Uhl[1,2], Tommaso Pavan[1,2], Malwina Molendowska[3], Derek K. Jones[3], Marco Palombo[3,4,], Ileana Jelescu[1,2,*]*

[1]Department of Radiology, Lausanne University Hospital (CHUV), Lausanne, Switzerland, [2]School of Biology and Medicine, University of Lausanne, Lausanne, Switzerland, [3]Cardiff University Brain Research Imaging Centre (CUBRIC), Cardiff University, Cardiff, United Kingdom, [4]School of Computer Science and Informatics, Cardiff University, Cardiff, UK, *joint senior authorship

Supplementary Material

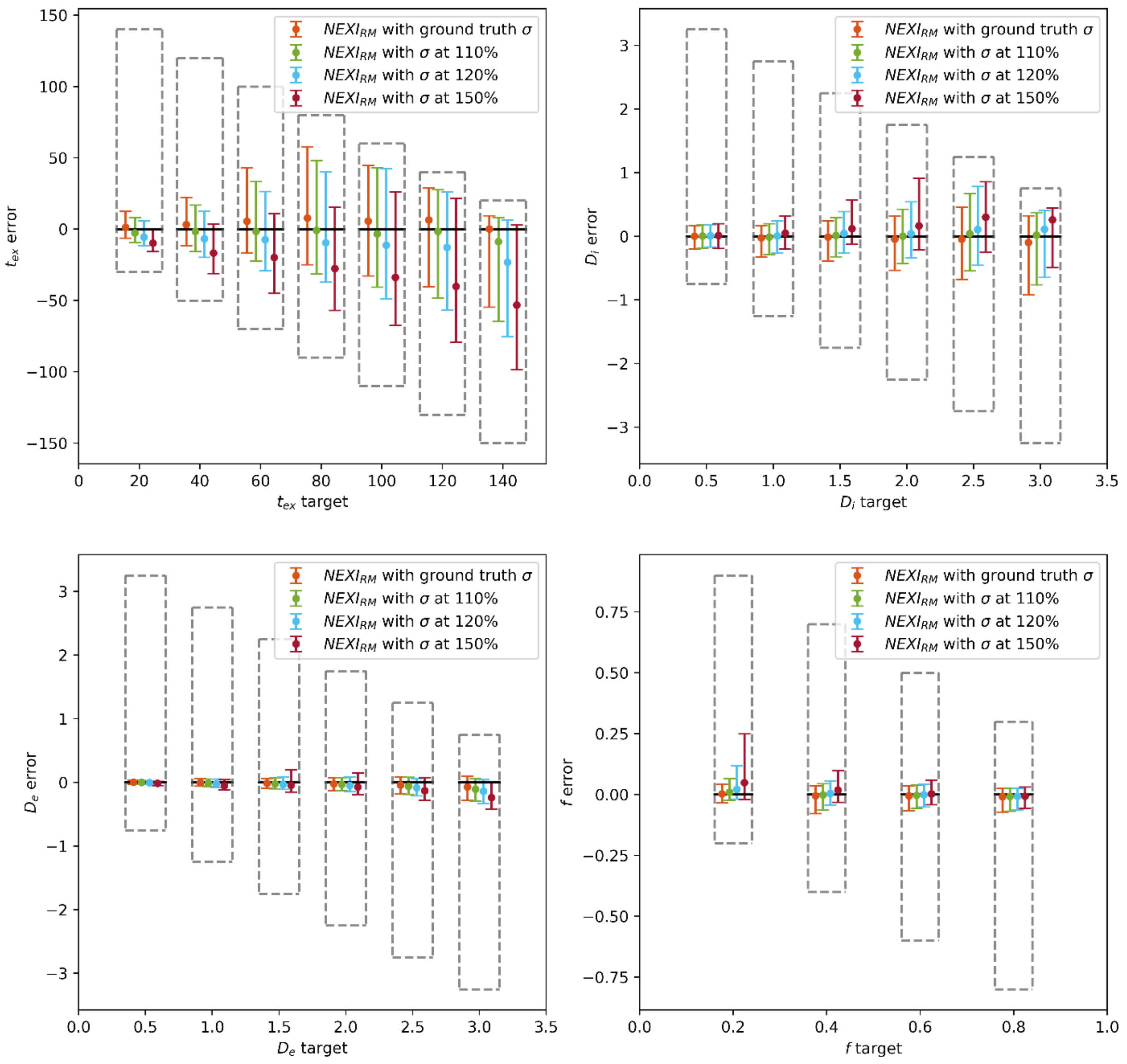

Figure S1. Boxplots (median and interquartile range) of parameter estimates by $NEXI_{RM}$ using the true and overestimated σ, on a synthetic dataset with random Rician noise (true σ=0.03). The error is defined as the difference between the estimation and the target value. The upper and lower limits of the grey dashed box represent the maximum and minimum error of the estimator, in the given bin, due to lower and upper bounds in the NLS algorithm.

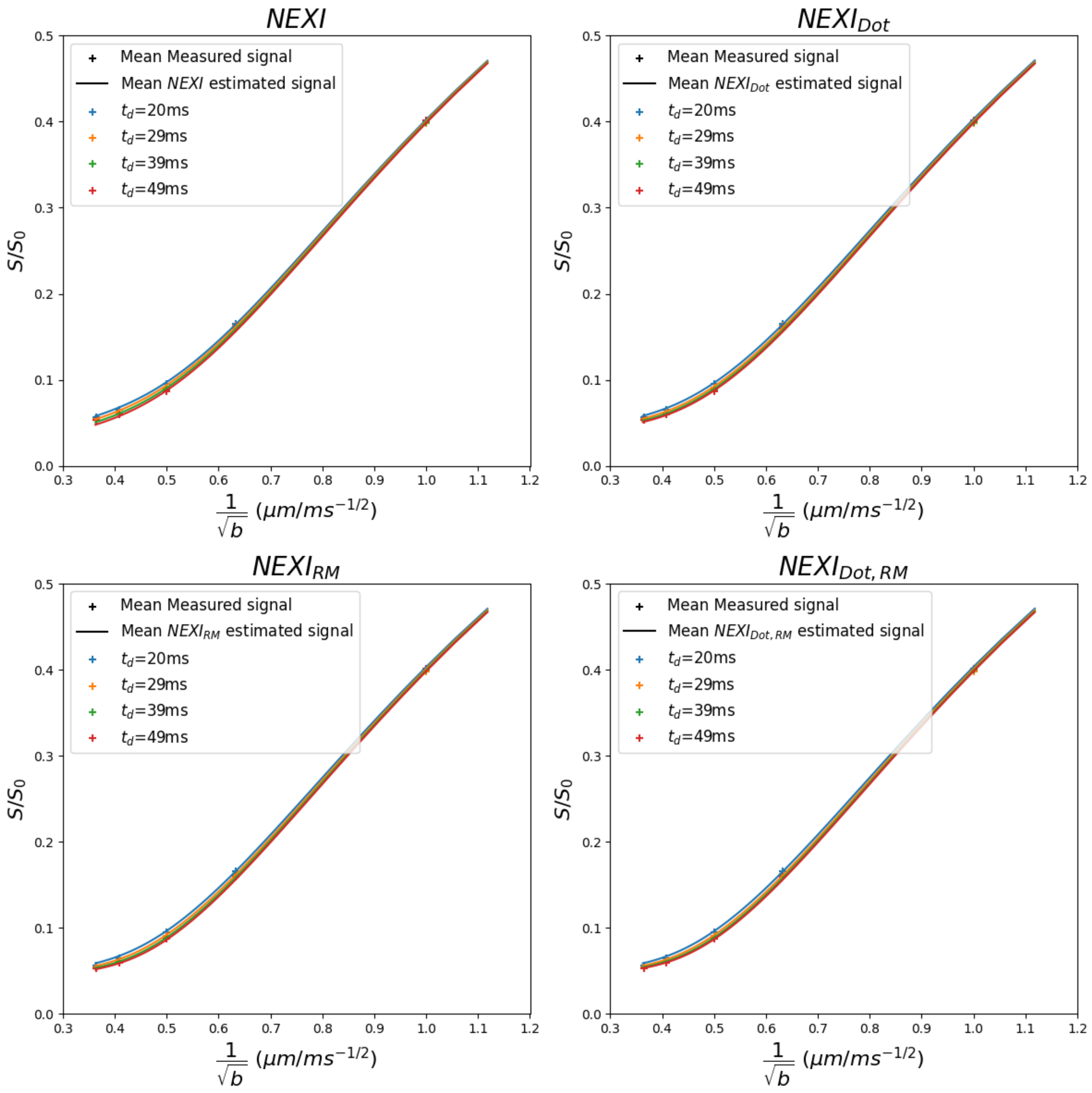

Figure S2. Mean estimated signal in the cortical ribbon by the four NEXI model variants at both high and low b-value compared to the mean measured signal, represented by plus signs. Each color represents a different diffusion time. The low b-value (especially at b=2.5ms/μm²) mean measured signal caused the estimated mean curve to shift upwards at high b-value.

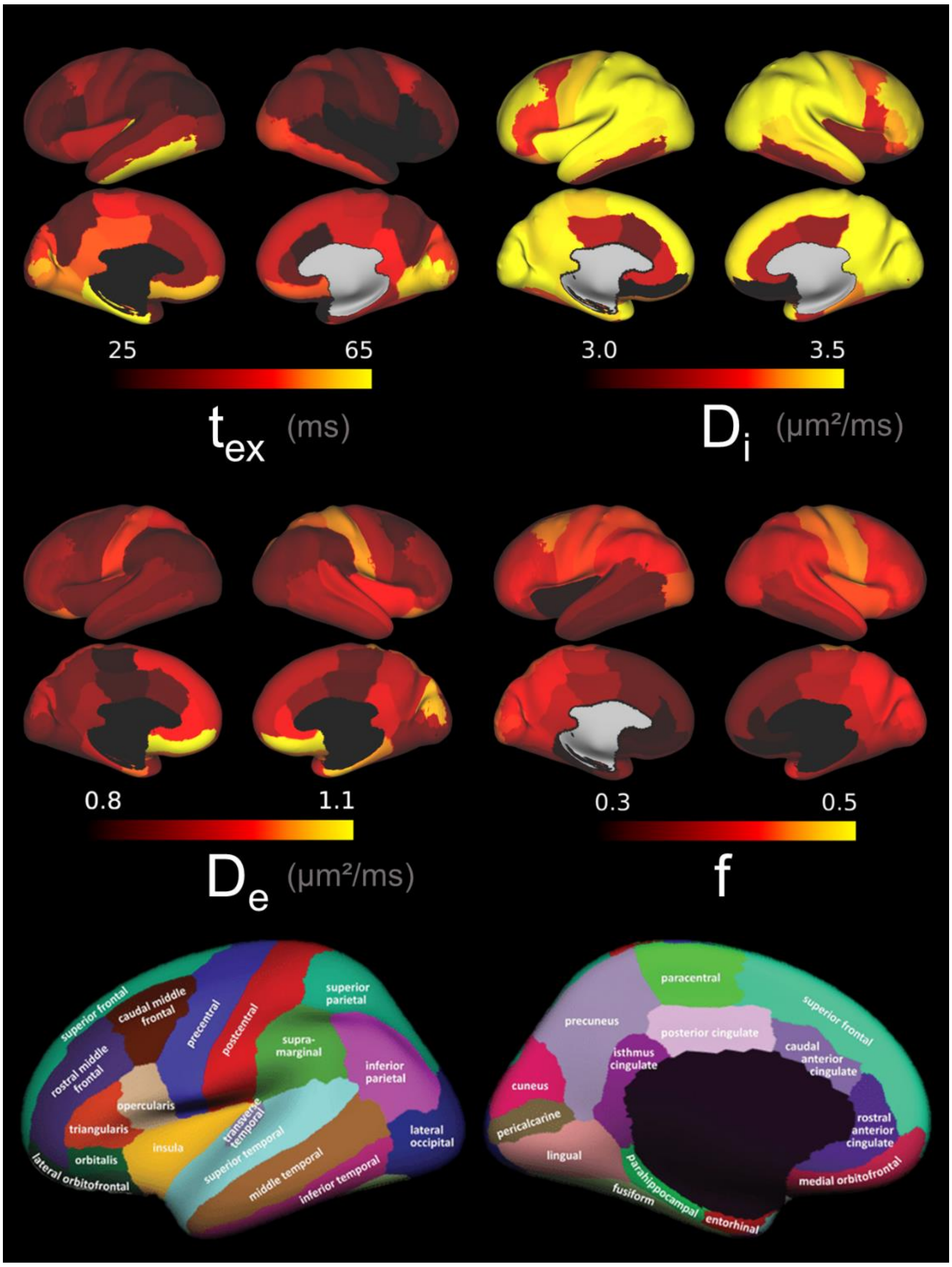

Figure S3. Average $NEXI_{RM}$ estimates for each DKT ROI. The averages were computed from the volumetric maps and projected to the study-average inflated cortical surface. We find patterns of a larger fraction of neurites in the motor cortex and premotor area. The exchange time is longer in the temporal and occipital lobes, and in the premotor area. A faster extracellular diffusivity is observed in the somatosensory cortex.

Figure S4. Bland-Altman plots on the t_{ex} and f estimations from NEXI (A-B), $NEXI_{dot}$ (C-D) and $NEXI_{dot,RM}$ (E-F) models. Each row and column refer to the same subject. On the diagonal, the two sessions of each subject are compared. In the upper triangle, the results of the first session of each subject are compared.

A.

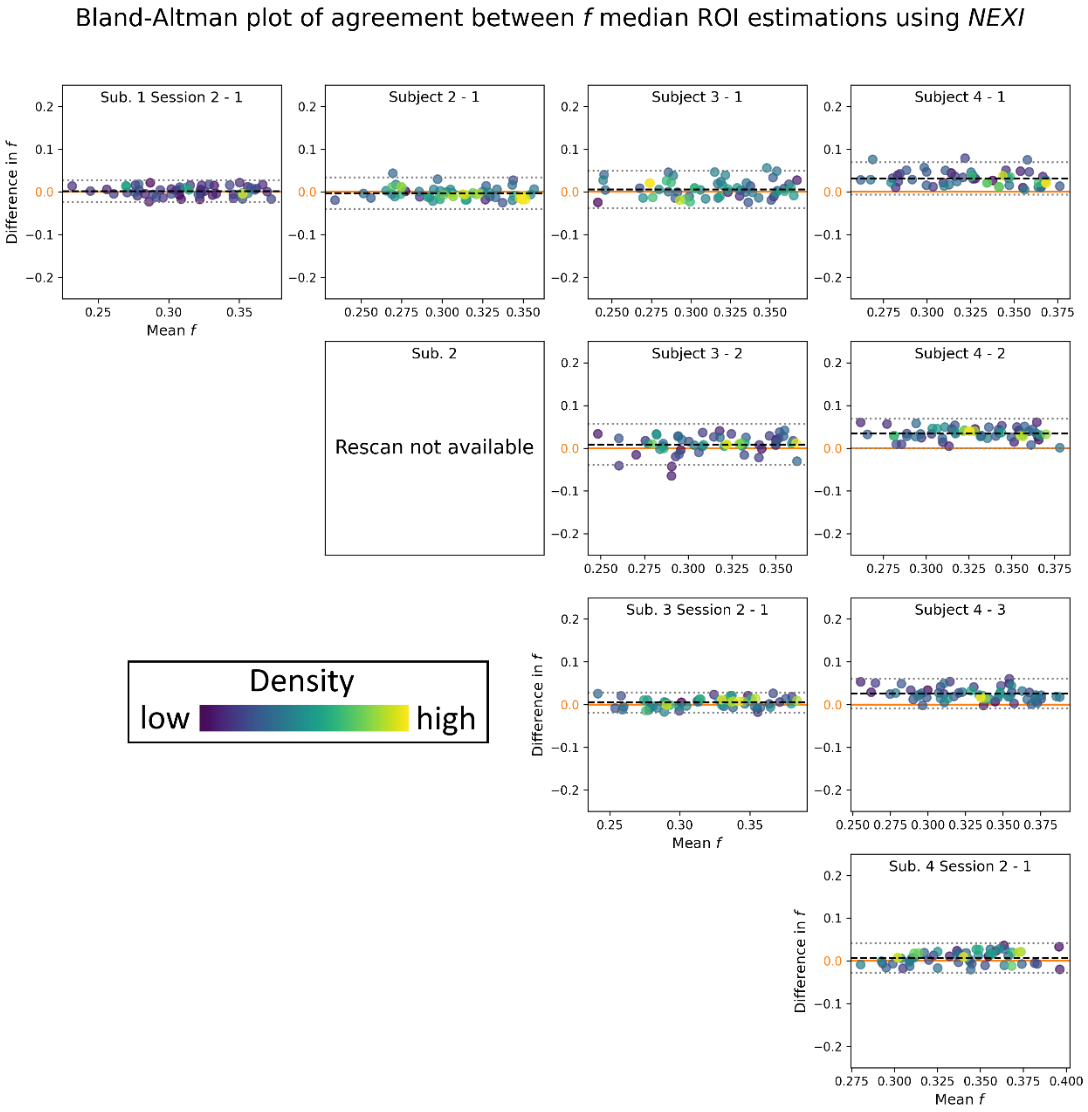

B.

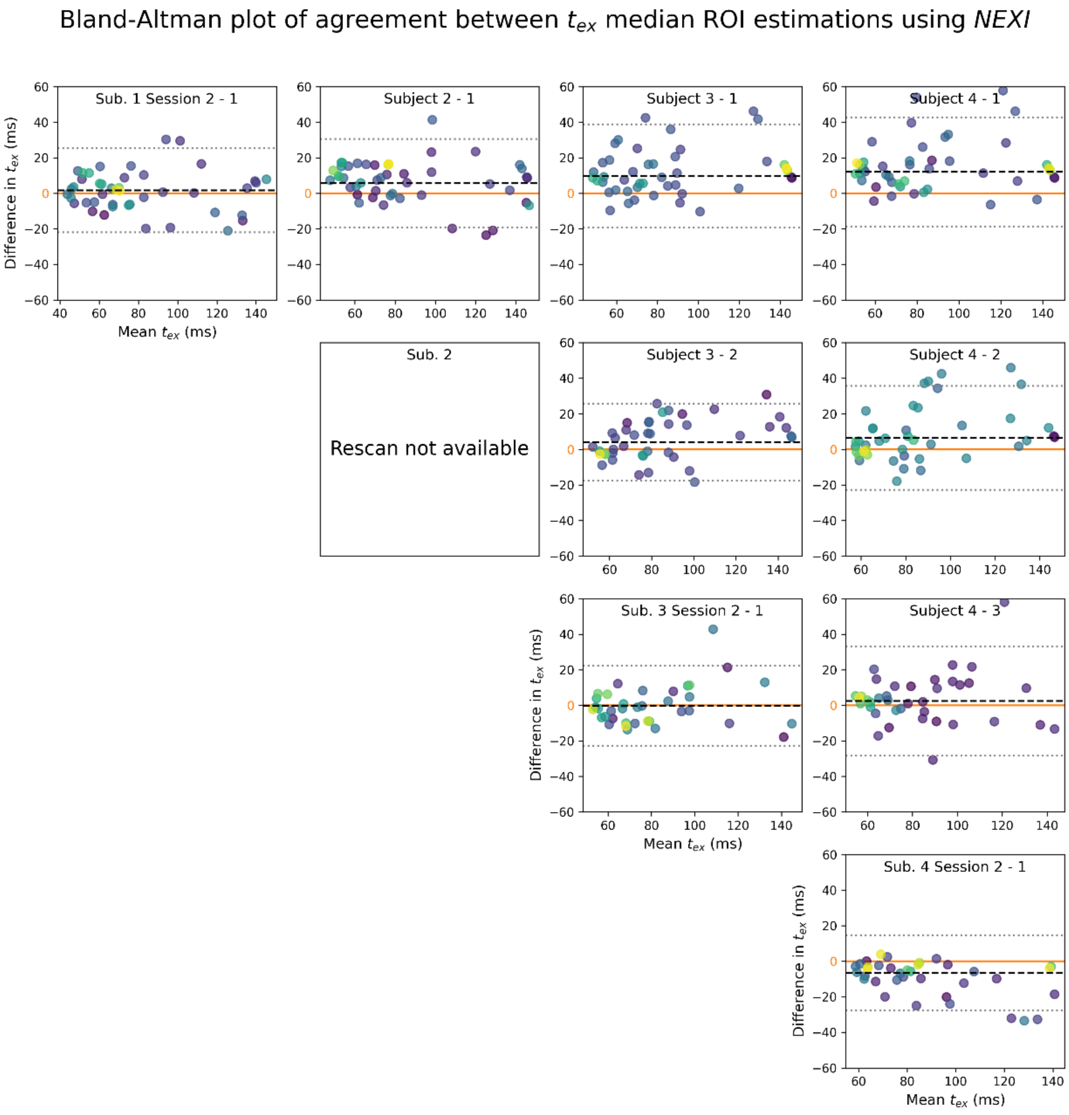
Bland-Altman plot of agreement between t_{ex} median ROI estimations using *NEXI*
Sub. 1 Session 2 - 1
Subject 2 - 1
Subject 3 - 1
Subject 4 - 1
Sub. 2
Rescan not available
Subject 3 - 2
Subject 4 - 2
Sub. 3 Session 2 - 1
Subject 4 - 3
Sub. 4 Session 2 - 1
Difference in t_{ex} (ms)
Mean t_{ex} (ms)

C.

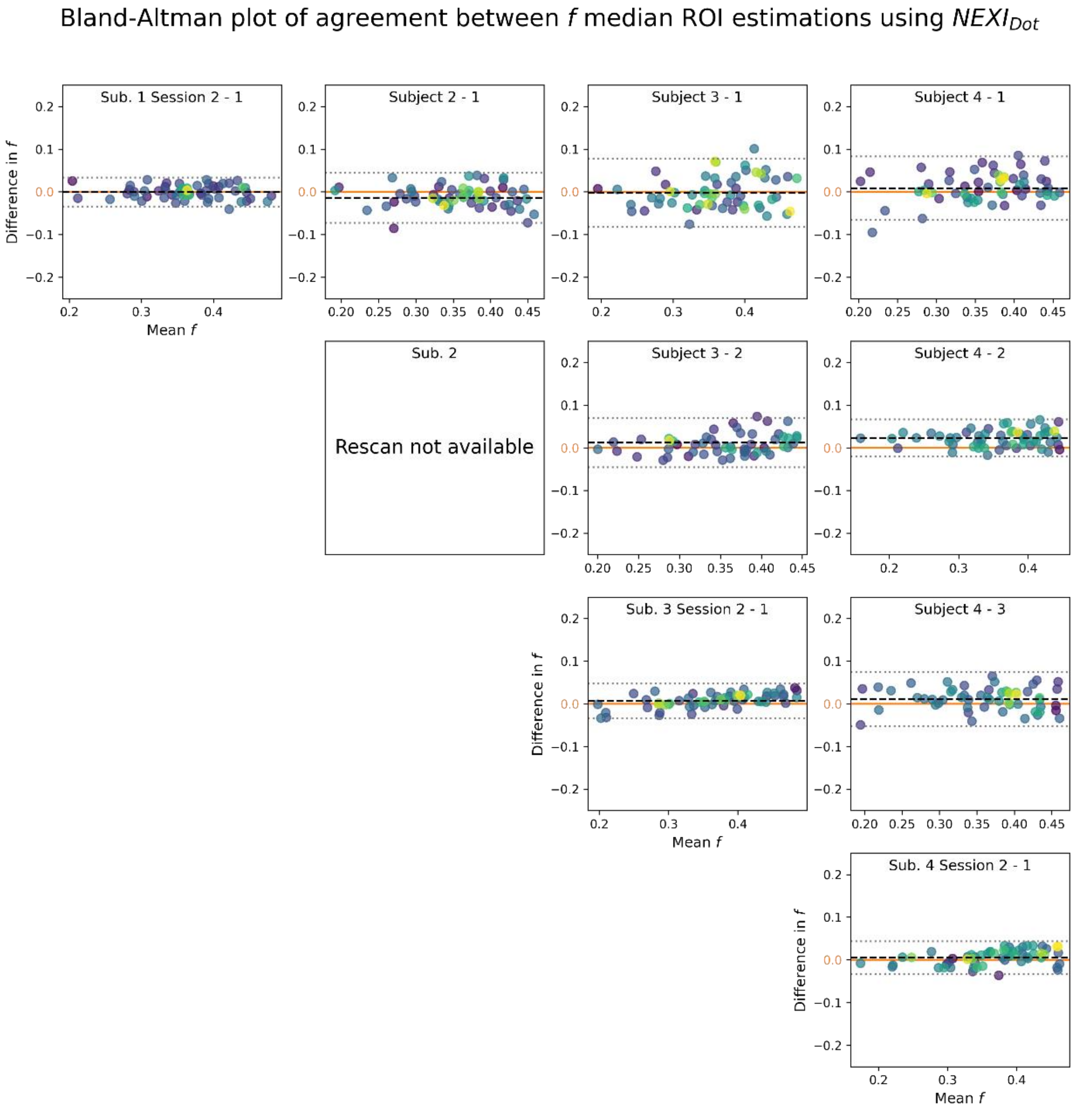

Bland-Altman plot of agreement between f median ROI estimations using NEXI_Dot
Sub. 1 Session 2 - 1
Subject 2 - 1
Subject 3 - 1
Subject 4 - 1
Sub. 2
Rescan not available
Subject 3 - 2
Subject 4 - 2
Sub. 3 Session 2 - 1
Subject 4 - 3
Sub. 4 Session 2 - 1
Difference in f
Mean f

D.

Bland-Altman plot of agreement between t_{ex} median ROI estimations using $NEXI_{Dot}$

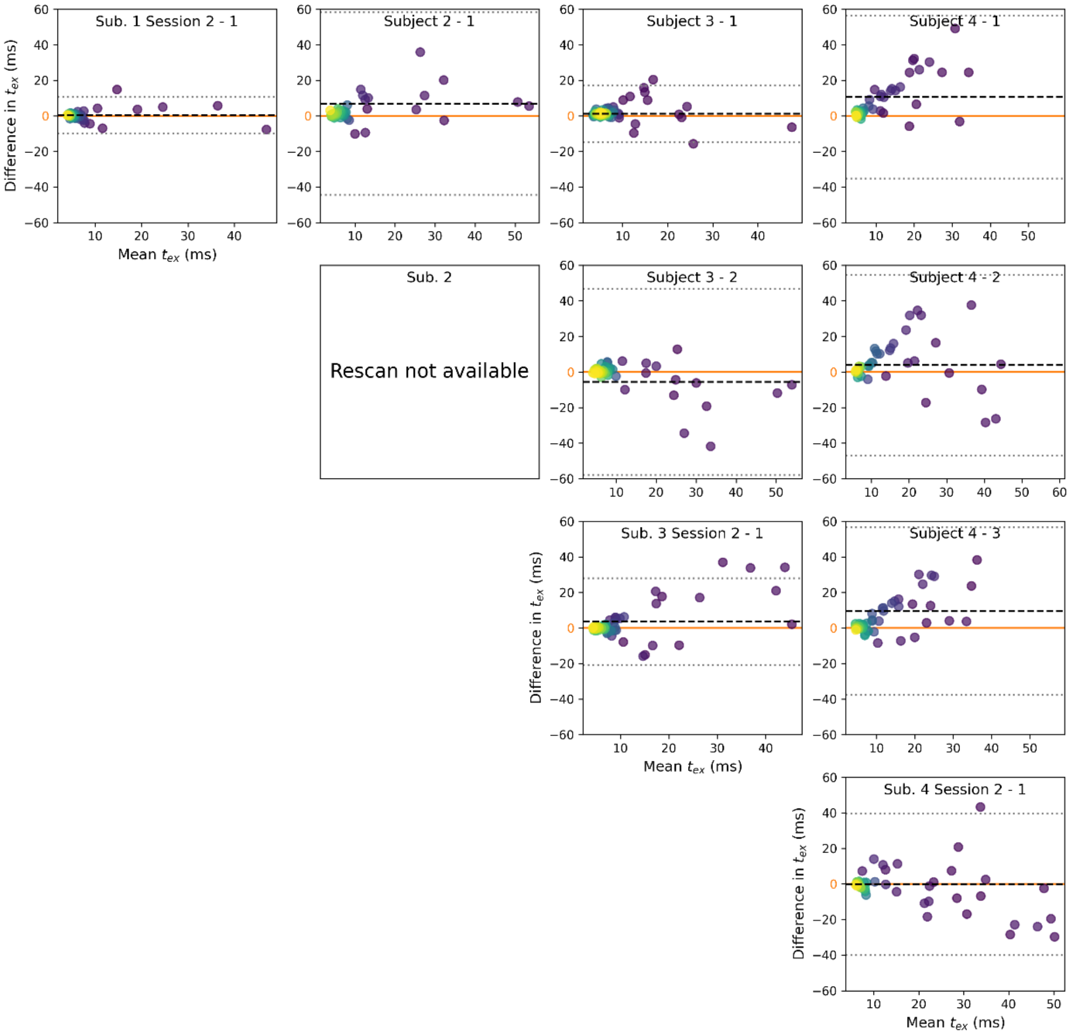

E.

Bland-Altman plot of agreement between f median ROI estimations using $NEXI_{Dot,RM}$

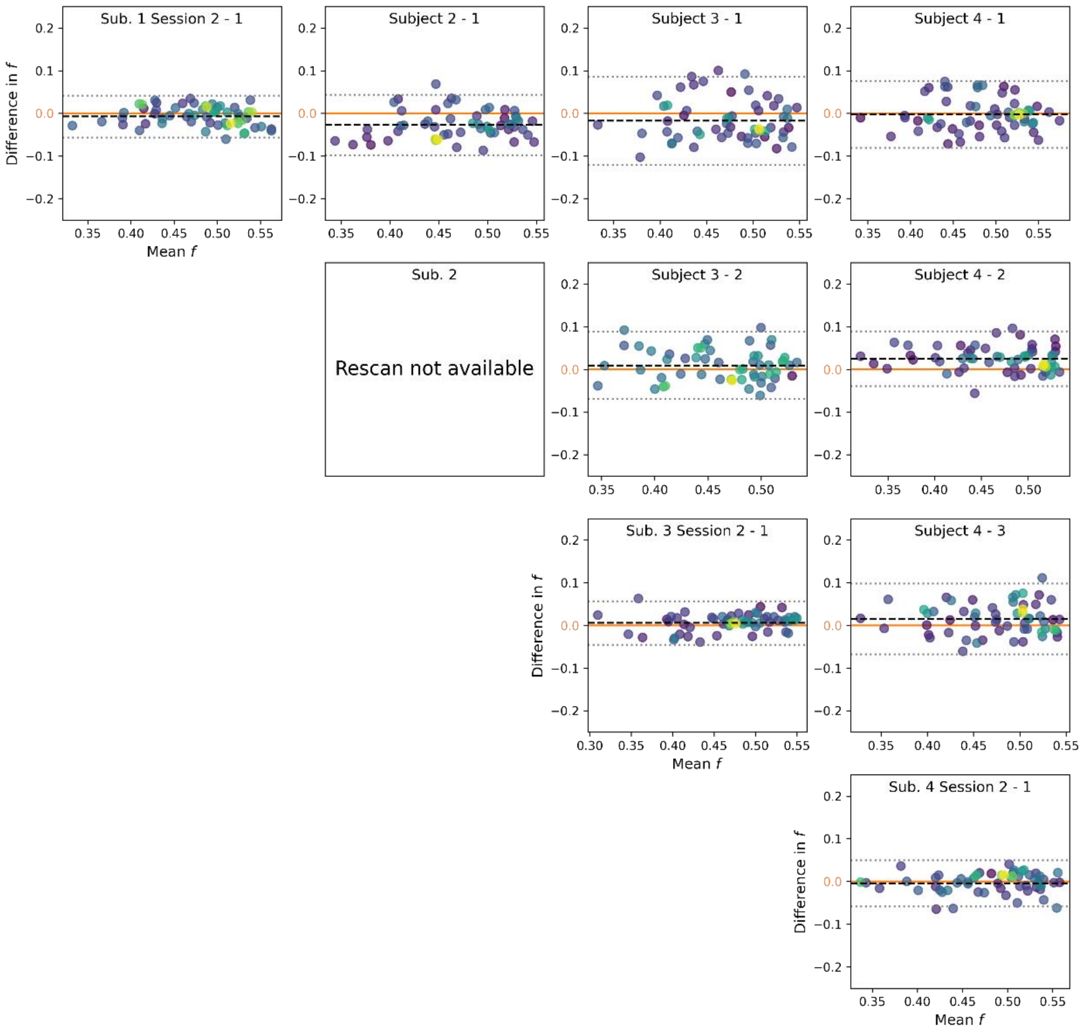

F.

Bland-Altman plot of agreement between t_{ex} median ROI estimations using $NEXI_{Dot,RM}$

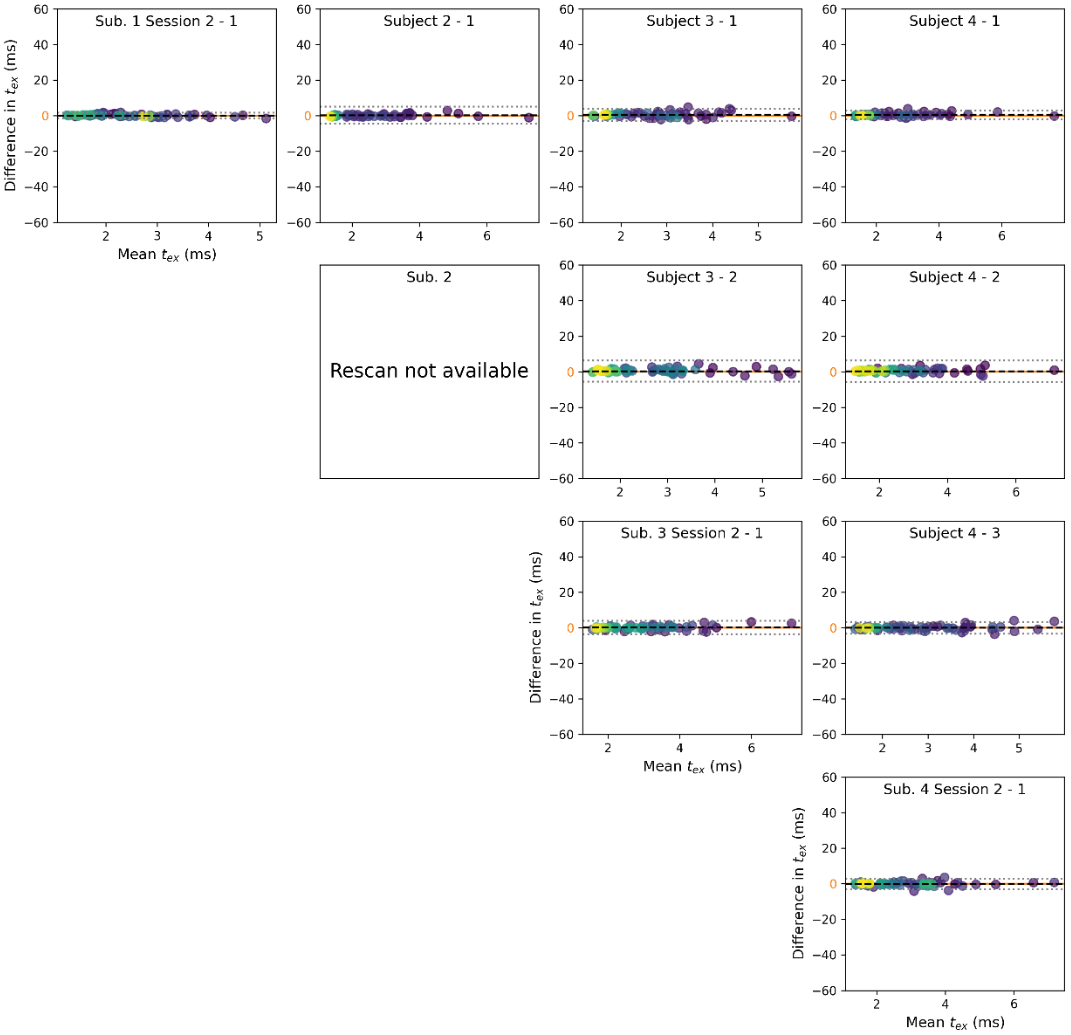

Figure S5. Bland-Altman plots on the D_i (A) and D_e (B) estimations from the $NEXI_{RM}$ model. Each row and column refer to the same subject. On the diagonal, the two sessions of each subject are compared. In the upper triangle, the results of the first session of each subject are compared.

A.

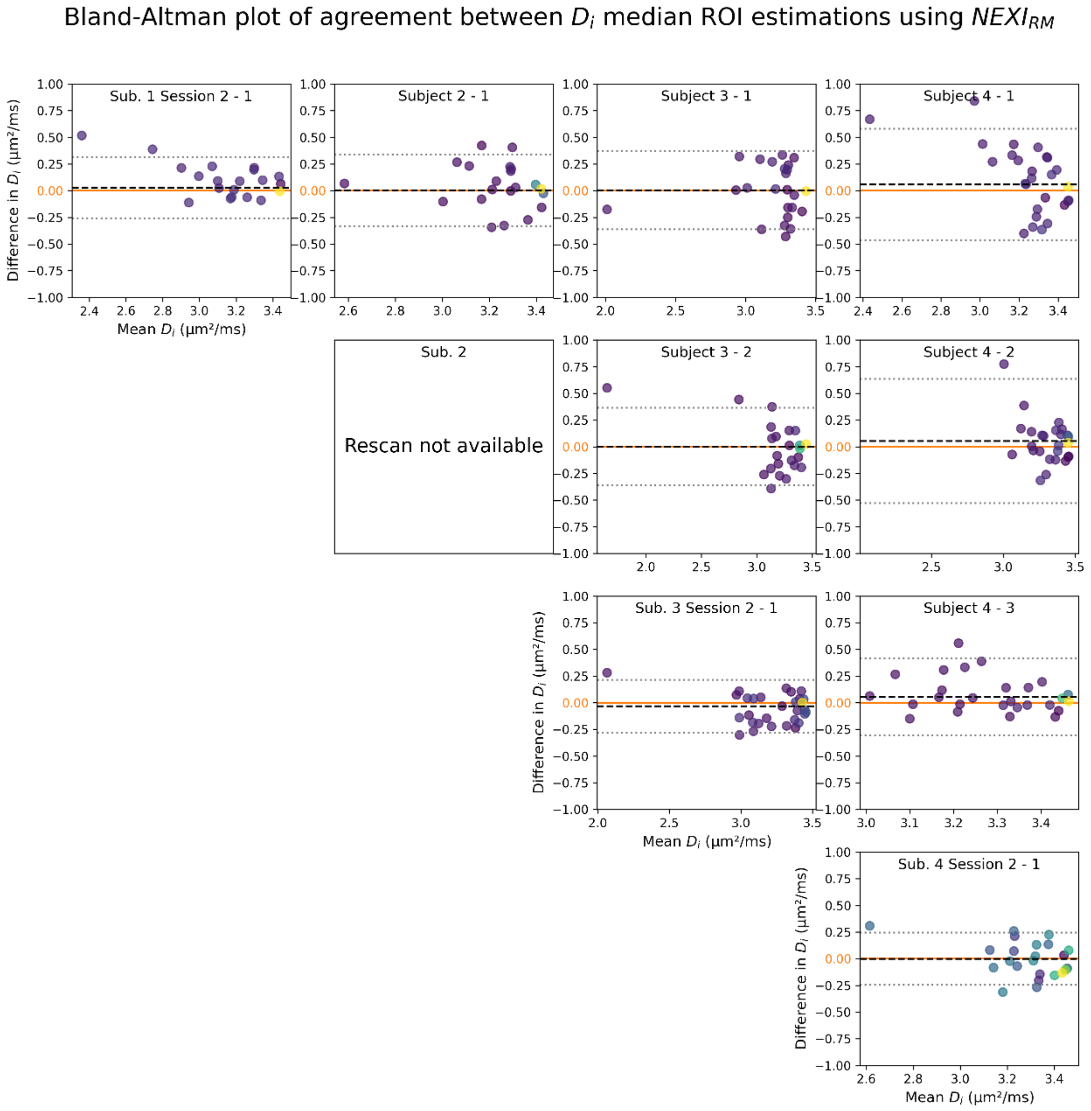

B.

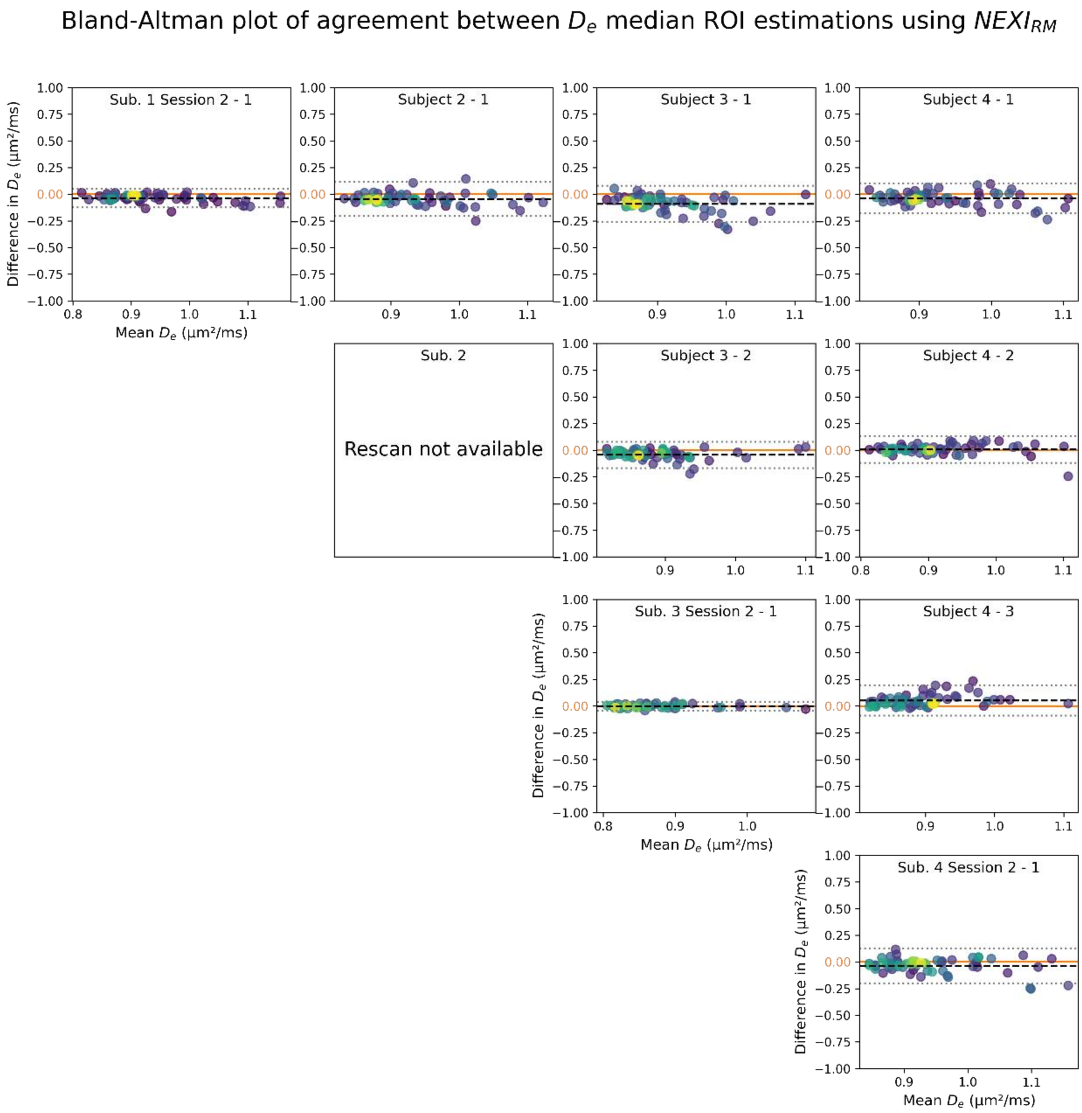
Bland-Altman plot of agreement between D_e median ROI estimations using $NEXI_{RM}$
Sub. 1 Session 2 - 1
Subject 2 - 1
Subject 3 - 1
Subject 4 - 1
Sub. 2
Rescan not available
Subject 3 - 2
Subject 4 - 2
Sub. 3 Session 2 - 1
Subject 4 - 3
Sub. 4 Session 2 - 1
Difference in D_e (µm²/ms)
Mean D_e (µm²/ms)
